# DNA methylation patterns in the frontal lobe white matter of multiple system atrophy, Parkinson’s disease, and progressive supranuclear palsy: a cross-comparative investigation

**DOI:** 10.1007/s00401-024-02764-4

**Published:** 2024-07-12

**Authors:** Megha Murthy, Katherine Fodder, Yasuo Miki, Naiomi Rambarack, Eduardo De Pablo Fernandez, Lasse Pihlstrøm, Jonathan Mill, Thomas T. Warner, Tammaryn Lashley, Conceição Bettencourt

**Affiliations:** 1 Queen Square Brain Bank for Neurological Disorders, UCL Queen Square Institute of Neurology, London, UK; 2 Department of Clinical and Movement Neurosciences, UCL Queen Square Institute of Neurology, London, UK; 3 Department of Neurodegenerative Disease, UCL Queen Square Institute of Neurology, London, UK; 4 Department of Neuropathology, Institute of Brain Science, Hirosaki University Graduate School of Medicine, Hirosaki, Japan; 5 Reta Lila Weston Institute, UCL Queen Square Institute of Neurology, London, UK; 6 Department of Neurology, Oslo University Hospital, Oslo, Norway; 7 Department of Clinical and Biomedical Sciences, Faculty of Health and Life Sciences, University of Exeter, Exeter, UK

**Keywords:** Parkinsonian disorders, Multiple system atrophy, Parkinson’s disease, Progressive supranuclear palsy, DNA methylation, EWAS, WGCNA

## Abstract

Multiple system atrophy (MSA) is a rare neurodegenerative disease characterized by neuronal loss and gliosis, with oligodendroglial cytoplasmic inclusions (GCIs) containing α-synuclein being the primary pathological hallmark. Clinical presentations of MSA overlap with other parkinsonian disorders, such as Parkinson’s disease (PD), dementia with Lewy bodies (DLB), and progressive supranuclear palsy (PSP), posing challenges in early diagnosis. Numerous studies have reported alterations in DNA methylation in neurodegenerative diseases, with candidate loci being identified in various parkinsonian disorders including MSA, PD, and PSP. Although MSA and PSP present with substantial white matter pathology, alterations in white matter have also been reported in PD. However, studies comparing the DNA methylation architectures of white matter in these diseases are lacking. We therefore aimed to investigate genome-wide DNA methylation patterns in the frontal lobe white matter of individuals with MSA (*n* = 17), PD (*n* = 17), and PSP (*n* = 16) along with controls (*n* = 15) using the Illumina EPIC array, to identify shared and disease-specific DNA methylation alterations. Genome-wide DNA methylation profiling of frontal lobe white matter in the three parkinsonian disorders revealed substantial commonalities in DNA methylation alterations in MSA, PD, and PSP. We further used weighted gene correlation network analysis to identify disease-associated co-methylation signatures and identified dysregulation in processes relating to Wnt signaling, signal transduction, endoplasmic reticulum stress, mitochondrial processes, RNA interference, and endosomal transport to be shared between these parkinsonian disorders. Our overall analysis points toward more similarities in DNA methylation patterns between MSA and PD, both synucleinopathies, compared to that between MSA and PD with PSP, which is a tauopathy. Our results also highlight several shared DNA methylation changes and pathways indicative of converging molecular mechanisms in the white matter contributing toward neurodegeneration in all three parkinsonian disorders.

**Supplementary Information:**

The online version contains supplementary material available at 10.1007/s00401-024-02764-4.

## Introduction

Multiple system atrophy is a rare adult-onset, rapidly progressing, neurodegenerative disorder characterized by neuronal loss and gliosis in multiple areas of the brain, brainstem, and spinal cord [57]. Diagnosing MSA in the early stages of the disease can be challenging owing to its overlapping clinical features with other parkinsonian disorders, such as Lewy body diseases (LBD) [i.e., Parkinson’s disease (PD) and dementia with Lewy bodies (DLB)] and progressive supranuclear palsy (PSP) [41, 43, 71]. Despite sharing several neuropathological underpinnings, particularly in the advanced stages of the disease, each condition exhibits distinct neuropathological hallmarks [46]. For instance, while both MSA and PD are synucleinopathies, MSA is uniquely characterized by the presence of glial cytoplasmic inclusions (GCIs) containing α-synuclein in oligodendrocytes, whereas PD is pathologically characterized by the presence of α-synuclein aggregates, mostly in neurons, known as Lewy bodies [20]. PSP on the other hand is a 4R tauopathy characterized by tau inclusions in the form of tufted astrocytes, neuronal tangles, and coiled bodies in oligodendrocytes, and therefore shares the common neuropathological feature of gliosis with MSA [31]. More interestingly, a co-existence of α-synuclein and tau has been observed, and both proteins share striking common characteristics suggesting a crosstalk between the two types of proteinopathies (i.e., synucleinopathies and tauopathies) and the involvement of common molecular mechanisms driving neurodegeneration [46]. White matter abnormalities, such as demyelination, axonal loss, and gliosis, have been documented in all three neurodegenerative parkinsonian disorders, with significant white matter involvement being observed in MSA [15], tau pathology extending to white matter regions in PSP [66], and recent studies pointing toward white matter changes in PD playing roles in disease development and progression [77].

The intricate mechanisms underlying neurodegeneration encompass a complex interplay between genetic, epigenetic or regulatory factors, along with environmental exposures. Several studies have delved into the molecular underpinnings of MSA, particularly within the white matter. One such study compared gray and white matter frontal cortex transcriptomes in MSA and control subjects [44], whereas another investigated the transcriptional profiles of cerebellar white matter in MSA [56]. Epigenetic mechanisms also play a pivotal role in the tissue- and cell type-specific changes that occur during disease development and progression. DNA methylation is one of the most commonly studied epigenetic mechanisms, and alterations in DNA methylation have been reported in several neurodegenerative disorders, including parkinsonian disorders [47].

Our group previously examined the effects of DNA methylation in white matter tissue from different brain regions in MSA compared to controls [6]. This study identified changes in key myelin and oligodendrocyte-related genes, including *MOBP*, as among the most differentially methylated loci in MSA. The increased DNA methylation of *MOBP* locus observed in MSA correspondingly showed lower mRNA expression levels in the cerebellar white matter and although the protein levels did not differ from controls, MOBP protein was found to be mislocalized into the GCIs in MSA [7]. Subsequently, another study revealed a shift from cytosine methylation toward hydroxymethylation in a locus mapping to the *AREL1* gene, as well as several immune system-related changes in the prefrontal cortex gray and white matter mixed tissue in MSA compared to controls [8, 60]. Multiple epigenome-wide association studies (EWAS) in Parkinson's disease (PD) have revealed significant DNA methylation changes in genes, such as *CYP2E1*, *SNCAIP*, and several others across various brain regions, implicating pathways, such as Wnt and Hippo, in PD pathology [13, 32]. Additionally, multiomics studies reinforce the role of DNA methylation in regulating PD risk genes, including *GPNMB*, *TMEM163*, and *CTSB* [33]. Similarly, an EWAS in the prefrontal lobe tissue of PSP individuals identified increased methylation in *DLX1*, a transcription factor influencing MAPT expression, which might contribute to PSP pathogenesis and another study revealed several DNA methylation changes in the vicinity of PSP-associated loci, including *MOBP* [3, 74].

Given the clinical overlap and shared pathogenetic mechanisms between parkinsonian disorders, such as MSA, PD, and PSP, a comparative analysis of DNA methylation profiles could help elucidate molecular changes common across diseases and identify alterations specific to each pathology. To date, no study has specifically investigated DNA methylation alterations in the white matter of PD or PSP. Although PD is primarily considered to be a gray matter disease, recent transcriptomic studies have revealed dysregulation of oligodendrocyte and myelin-related genes, and a loss of oligodendrocytes in post-mortem midbrain tissue of PD patients [77]. Conversely, PSP is characterized by abnormal tau protein aggregation in both gray and white matter regions. Single-nucleus RNA sequencing in the subthalamic nucleus of PSP identified specific contributions of the glial cell types including increased EIF2 signaling, in addition to dysregulation in genes and pathways related to apoptotic regulation and autophagy signaling in astrocytes and oligodendrocytes [22, 75]. As in the case of MSA, bulk transcriptomic analysis also revealed changes in gene expression of myelin-related genes in PSP [4]. Additionally, genetic variants in *MOBP* have been associated with PSP risk [11, 28, 61]. Oligodendrocytes are one of the major cell types in the white matter (up to 75%) that contribute to the formation of myelin sheaths and have been time and again shown to play important roles in several neurodegenerative disease mechanisms [21, 52]. Moreover, DNA methylation patterns suggest accelerated epigenetic aging in these cells, likely resulting from a greater vulnerability of oligodendrocytes to aging [48, 49], which might, at least in part, be contributed by changes in DNA methylation.

To directly compare DNA methylation alterations in MSA white matter with those in PD and PSP, we focused on the frontal lobe, a region that is moderately affected in MSA, which also shows substantial involvement in PSP, as well as in the advanced stages (Braak stages 5 and 6) in PD. Additionally, previous data from our group has demonstrated a considerable overlap in DNA methylation alterations in MSA between the cerebellum (severely affected in MSA) and frontal lobe (moderately affected in MSA) [6]. Therefore, the primary objective of this study was to perform a cross-comparative genome-wide DNA methylation analysis in the frontal lobe white matter of MSA, PD, and PSP to identify distinct and shared DNA methylation alterations, and to elucidate mechanisms determining the vulnerability of specific cell types, particularly the oligodendrocytes, to dysfunction and/or protein aggregation in the different diseases.

## Materials and methods

### Human post-mortem brain tissues and their clinical and demographic characteristics

All post-mortem human brain tissues for the primary cohort were obtained from the UCL Queen Square Institute of Neurology Queen Square Brain Bank, with ethical approval for both brain donation and research protocols granted by the NRES committee—London central. The cohort was composed of human post-mortem brain tissues from individuals diagnosed with three neurodegenerative parkinsonian disorders, MSA (*n* = 17), PD (*n* = 17), PSP (*n* = 17) and neurologically healthy controls (*n* = 17). Disease history, neuropathological findings, age, and sex were characterized for all disease cases and controls. In addition, data from the primary cohort were compared to data previously generated by our group [6], which included white matter tissues from the cerebellum of individuals with MSA (*n* = 41) and controls (*n* = 21), as well as other publicly available datasets comprising gray and white matter mixed tissue from the prefrontal cortex of individuals with MSA (*n* = 39) and controls (*n* = 37) (GSE143157) [60], and of PSP cases (*n* = 93) and controls (*n* = 70) (GSE75704)[74]. Another dataset comprising gray matter from the frontal cortex of LBD cases (PD = 60; PD with dementia = 60; DLB = 15) and controls (*n* = 68) (GSE203332) [55], from the Netherlands Brain Bank (NBB) was also used. Samples with Alzheimer’s disease (AD) or mixed AD pathology, samples with incidental LBD, samples with thal amyloid phase ≥ 4, and samples with Braak neurofibrillary tangle staging ≥ 4, were removed prior to analysis of the LBD dataset. Demographic characteristics for all cohorts are detailed in Table 1. GCI burden in MSA was assessed by a neuropathologist (Y.M.) using α-synuclein immunohistochemical staining in the frontal lobe white matter. The density of GCIs was graded using a modified grading scale as described previously: 0: 0–5 inclusions; 1 + : 6–20 inclusions; 2 + : 21–40 inclusions; and 3 + : ≥ 41 inclusions [42]. Ten areas were randomly selected in each case, and the density of GCIs was assessed using a × 20 objective. Average GCI counts across the 10 areas were also used.

**Table 1:** Demographic characteristics of the cohorts [6, 55, 60, 74]

Group	Sample no	Mean age (SD)	Sex	
			Female	Male
MSA, PD, and PSP frontal lobe white matter				
CTRL	17	72.35 (4.47)	9 (53%)	8 (47%)
MSA	17	67.71 (6.47)	8 (47%)	9 (53%)
PD	17	68.06 (3.67)	9 (53%)	8 (47%)
PSP	17	65.35 (3.75)	8 (47%)	9 (53%)
MSA cerebellar white matter				
CTRL	21	80.29 (9.05)	10 (48%)	11 (52%)
MSA	41	64.34 (7.9)	20 (49%)	21 (51%)
MSA prefrontal cortex gray and white matter				
CTRL	37	72.97 (10.45)	18 (49%)	19 (51%)
MSA	39	66.03 (5.83)	22 (56%)	17 (44%)
LBD frontal cortex gray matter				
CTRL	68	80.41 (11.16)	49 (72%)	19 (28%)
DLB	15	76.73 (8.39)	3 (20%)	12 (80%)
PD	60	76.70 (8.93)	27 (45%)	33 (55%)
PDD	60	78.10 (5.85)	16 (27%)	44 (73%)
PSP prefrontal lobe gray and white matter				
CTRL	70	76.17 (7.93)	25 (42%)	45 (58%)
PSP	93	71.16 (5.32)	39 (42%)	54 (58%)

*CTRL* controls, *MSA* multiple system atrophy, *PD* Parkinson’s disease, *PSP* progressive supranuclear palsy, *LBD* Lewy body diseases, *DLB* Dementia with Lewy bodies, *PDD* PD with dementia

### Frontal lobe white matter DNA methylation profiling and data quality control

White matter (~ 100 μg) was carefully dissected from frozen frontal lobes (Brodmann area 9) of individuals with MSA, PD, PSP as well as neurologically healthy controls and genomic DNA was extracted using a standard phenol–chloroform–isoamyl alcohol method [39]. A total of 750 ng of DNA was subjected to bisulfite conversion using the EZ DNA Methylation kit (Zymo Research, Irvine, USA). Genome-wide DNA methylation was then performed using the Infinium HumanMethylationEPIC Bead Chip (Illumina). The resulting raw intensity (.idat) files were imported into R and subjected to thorough and stringent pre-processing and quality control checks using bioconductor packages, such as minfi [5], ChAMP [64], and WateRmelon [54]. Briefly, samples were evaluated by visualizing raw intensities and performing outlier detection based on pcount and interquartile ranges to remove poorly performing samples; additionally, samples with a high rate of failed probes (≥ 2%), a mismatch in the predicted versus phenotypic sex, and samples clustering separately in the multidimensional scaling were also excluded. Probes were filtered out if they mapped to the X or Y chromosomes, were cross-reactive, of poor quality, aligned to multiple locations, or included common genetic variants. Beta-Mixture Quantile (BMIQ) normalization method was applied to normalize the beta values, and M-values were computed as the logit transformation of beta values, as we previously described [6].

### DNA methylation-based deconvolution of cell type proportions

Similar to other types of genomic data, DNA methylation data derived from bulk tissue is susceptible to biases arising from variations in the cellular makeup. To address this issue, we utilized the recently developed R package ‘CEll TYpe deconvolution Goodness’ (CETYGO) [62, 70]. Building upon the functionalities of the deconvolution algorithm in the minfi package, CETYGO incorporates estimations of relative proportions of neurons (NeuN +), oligodendrocytes (SOX10 +), and other glial brain cell types (Double − [NeuN − /SOX10 −]) based on reference data obtained from fluorescence-activated sorted nuclei from cortical brain tissue [62]. This enabled us to estimate the cell type proportions from the frontal lobe white matter DNA methylation profiles. Comparisons between the proportions of different cell types were carried out using the Kruskal–Wallis test with a significance threshold of *p*-value < 0.05.

### Differential DNA methylation analysis

Given the enhanced statistical robustness of M values [18], we employed M values for our linear regression models using the limma package to detect differentially methylated CpG sites in MSA, PD, and PSP relative to controls, as well as between disease comparisons. To account for potential confounding factors, we incorporated age, sex, post-mortem interval (PMI), neuronal (NeuN +) proportions, and proportions of glial cell types other than oligodendrocytes (Double − [NeuN − /SOX10 −]) as covariates into the model, along with technical variables (i.e., slide, and array). The abovementioned covariates were associated with the first 5 principal components (PCs), and PCs beyond the 5th PC explained < 5% of the overall variance. Additionally, we utilized the SVA package [38] to estimate possible surrogate variables (SVs) and identify any unknown, latent, or unmodelled sources of noise; however, no SVs were detected using the above mentioned regression model. Adjusted beta and M values were obtained after adjusting for the covariates included in the model described above. A false discovery rate (FDR) adjusted *p*-value of < 0.05 was considered statistically significant at the genome-wide level, and unadjusted *p*-values ≤ 1 × 10^–5^ were considered suggestively significant. Adjusted beta values for CpGs that showed unadjusted *p*-values < 0.0001 were used to generate heatmaps for comparisons of diseases with controls as well as comparisons between diseases. We selected a delta beta value ≥ 5% as the threshold for identifying differentially methylated sites. This cutoff was chosen based on its established relevance in the literature to ensure the detection of robust biologically significant changes that are unlikely to arise due to technical variability or noise, as well as to minimize false positives.

### Weighted gene co-methylation network analysis (WGCNA)

To identify clusters of highly correlated methylation sites and to determine whether the correlation patterns were shared between the three neurodegenerative parkinsonian diseases, we used a systems biology method based on weighted gene correlation network analysis (WGCNA) to construct co-methylation networks [36]. We used the adjusted M values as input for this analysis. To minimize the influence of age differences between groups that arose after sample quality control, probes that were associated with age (unadjusted *p*-value < 0.01) were removed. Following this, top 10% CpGs mapping to genes that showed the highest variance across individuals regardless of their disease status were used as input (*n* = 53,032 CpGs). Sample clustering identified 5 outliers (1 MSA, 1 PSP, 2 PD, and 1 control), which were excluded, leaving a total of 60 samples for subsequent network analysis. A signed co-methylation network was generated using the function ‘blockwiseModules’, with the ‘mergeCutHeight’ set to 0.1, soft-thresholding power of 12, and minimum module size of 200. CpGs inside each module were represented by a weighted average termed the module eigengene (ME), and highly correlated modules (ME correlation > 0.75) were merged. Module membership (MM), defined as the Pearson correlation between the probe DNA methylation levels and each module eigengene value, represents the strength of association between a probe and its designated module. Additionally, we employed the applyKMeans function of the CoExpNets package [10] to reassign the MM. Gene significance (GS) was calculated as a function *GS* that assigns a non-negative number to each probe; and higher GS for a given probe indicates higher biological relevance of this probe to the trait or disease being considered. Within the disease-associated modules, we ranked genes based on their MM, prioritizing top hub genes using the function ‘chooseTopHubInEachModule’, which returns the probe with the highest connectivity in each module, looking at all probes in the methylation file [36]. Co-methylation networks were also produced in a similar way for the publicly available MSA cerebellar, MSA prefrontal cortex, and LBD and PSP datasets mentioned above, using a soft-thresholding power of 14 for the LBD dataset and 12 for all other datasets [6, 55, 60, 74].

### Module preservation analysis in additional datasets

To assess whether the modules identified in the frontal lobe white matter were also preserved in the additional datasets generated from different brain regions and comprising varying cell type compositions across the three neurodegenerative diseases, we employed preservation analysis [37]. We evaluated the module preservation for the modules identified in our data against data from previous studies for MSA cerebellar white matter, MSA prefrontal cortex gray and white matter mixed tissue, LBD frontal cortex gray matter, and PSP prefrontal lobe gray and white matter mixed tissue (Table 1). Preservation was determined using the ‘modulePreservation’ function of the WGCNA package with 200 permutations, and a Z-summary statistic was computed indicating high (Z-summary > 10), moderate (Z-summary 2–10), and no preservation (Z-summary < 2) of the module in the other datasets.

### Cell type enrichment and functional network analyses for the disease-associated co-methylation modules

To delve deeper into the cellular underpinnings of the disease-associated modules, cell type enrichment analysis was conducted. This analysis sought to identify whether the genes within each co-methylation module were enriched for markers of a specific cell type. Leveraging the EWCE package [63] and its accompanying single-cell mouse transcriptomic dataset [78], the enrichment analysis employed *p*-values derived from 10,000 iterations to pinpoint enriched cell types. Subsequently, gene lists were curated for the disease-associated modules enriched for oligodendrocytes by including genes with MM greater than 0.4, and functional module detection and enrichment analysis specific to the frontal lobe were performed using HumanBase (https://hb.flatironinstitute.org/) [25].

## Results

### Frontal lobe white matter cell type composition across neurodegenerative parkinsonian disorders

**Fig. 1:**
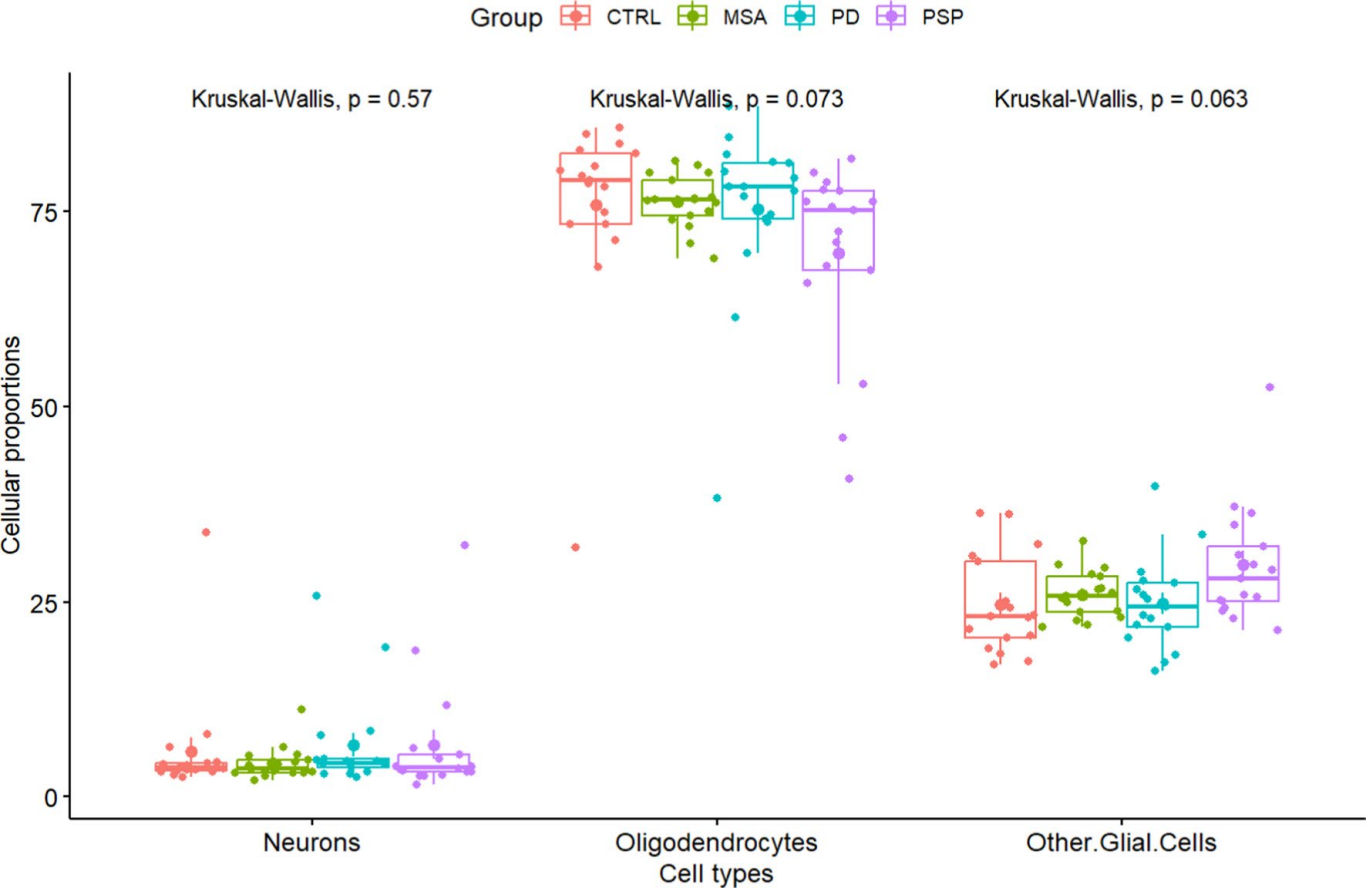
Cell type proportion estimates for the frontal lobe white matter tissue used for DNA methylation profiling. *CTRL* controls, *MSA* multiple system atrophy, *PD* Parkinson’s disease, *PSP* progressive supranuclear palsy. Comparisons between the different sample groups were carried out using the Kruskal–Wallis test with a significance threshold of *p*-value < 0.05

Following our previous DNA methylation study on MSA [6], we sought to compare methylation patterns across a range of neurodegenerative parkinsonian disorders. We analyzed the DNA methylation profiles generated from frontal lobe white matter from post-mortem brains of individuals with MSA, PD, PSP, and neurologically healthy controls. Following stringent quality control and filtering procedures, 65 samples (MSA = 17, PD = 17, PSP = 16, and CTRL = 15) and 734,360 probes were retained for further downstream analysis. Cell type deconvolution methods were employed to estimate the brain cell type proportions, confirming that the tissue samples were highly enriched for glial cells, particularly oligodendrocytes (average ~ 74% across groups), consistent with the expected composition of white matter (Fig. 1). Notably, in MSA and PSP, which exhibit pathological hallmarks in oligodendrocytes, we observed slightly lower proportions of oligodendrocytes, and corresponding higher proportions of other glial cell types in these two diseases compared to the other groups. However, such variations in cell type proportions failed to reach statistical significance across groups. We note that this lack of a significant drop in oligodendrocyte proportions in MSA and PSP may be due, in part, to the limited sensitivity of the Kruskal–Wallis test.

### Frontal lobe white matter DNA methylation profiling shows shared patterns across neurodegenerative parkinsonian disorders

**Fig. 2:**
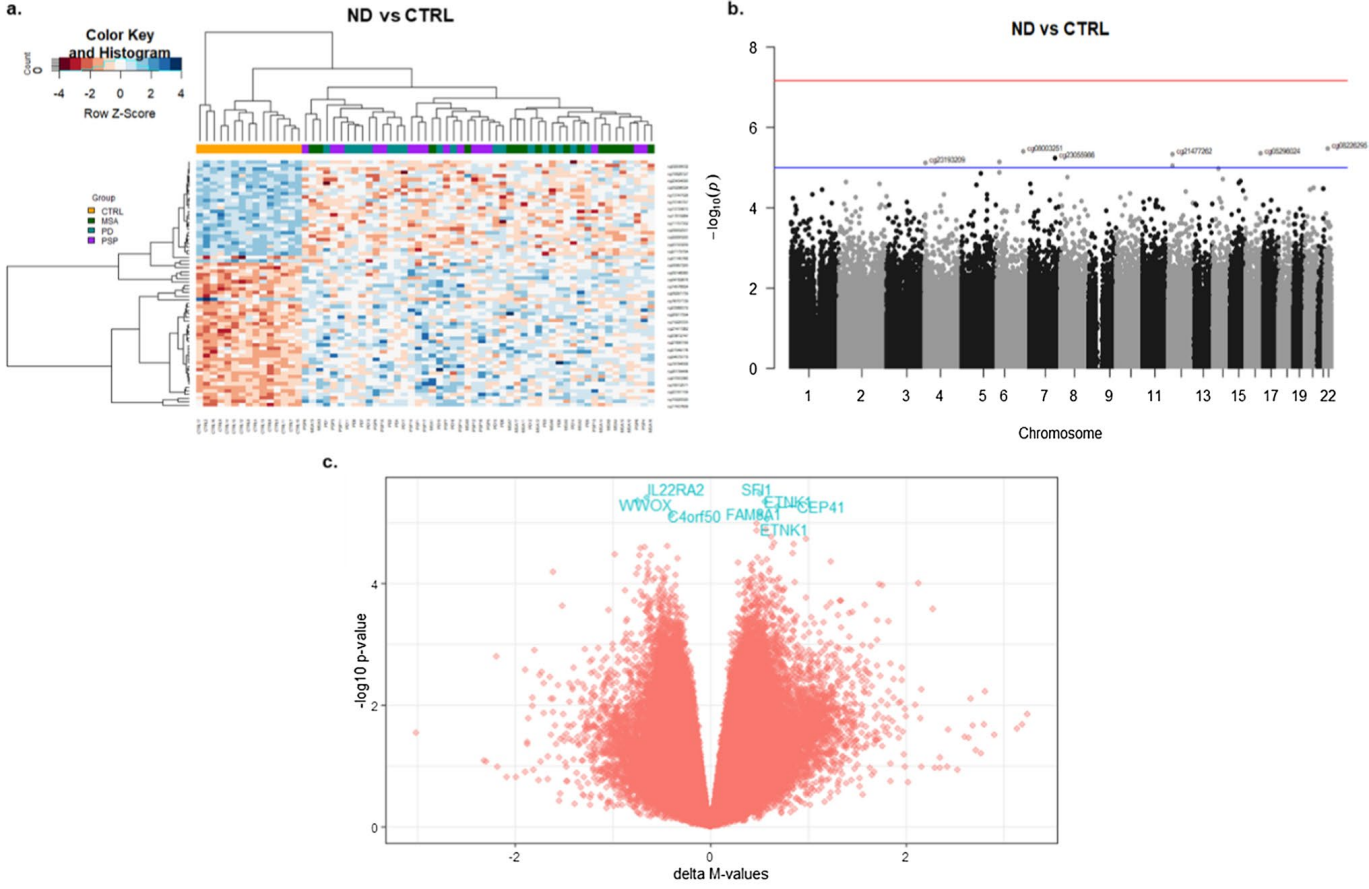
DNA methylation alterations in the frontal lobe white matter shared across the three neurodegenerative parkinsonian disorders (MSA, PD, and PSP). **a** Heatmap of the topmost differentially methylated loci (unadjusted *p* < 0.0001) identified in the neurodegenerative parkinsonian disorders compared to controls. The rows represent CpGs, columns represent samples, and the colors represent the direction as well as the magnitude of effect (adjusted β values) in all the samples (darker colors indicate larger effect sizes). **b** Manhattan plot showing suggestive associations between single DNA methylation sites (CpGs) and the neurodegenerative parkinsonian disorders. The red line indicates genome-wide significance threshold based on Bonferroni-corrected *p*-values (*p* = 6.8 × 10^–8^), and the blue line indicates a less stringent suggestive significance threshold of unadjusted *p* = 1 × 10^–5^. **c** Volcano plot showing the differentially methylated probes shared across the neurodegenerative parkinsonian disorders, gene names of CpGs with suggestive significance threshold of unadjusted *p* < 1 × 10^–5^ are highlighted in blue. *CTRL* controls, *ND* Neurodegenerative parkinsonian disorders

We next conducted an epigenome-wide association study (EWAS) using a linear regression model that accounted for age at death, sex, cellular proportions, and other covariates as detailed in the methods. Quantile–quantile plots showed no evidence of genomic inflation for any of the comparisons (Supplementary Fig. S1). When considering the topmost differentially methylated sites (*p* < 0.0001) in all three neurodegenerative parkinsonian disorders (MSA, PD, and PSP together) versus controls (Table S1), a clear separation was observed between the parkinsonian disorders and controls (Fig. 2a). However, little or no separation was observed within the three disease groups. Although not passing genome-wide significance after multiple testing corrections, eight CpGs mapping to seven genes (*SFI1*, *IL22RA2*, *WWOX*, *ETNK1*, *CEP41*, *FAM8A1*, and *C4orf50*) showed shared differential methylation (hypo- or hypermethylation) with a suggestive significance of unadjusted *p* < 1 × 10^–5^ across all diseases and, interestingly, several of these genes have been previously associated with neurological conditions (Fig. 2b,c, Table 2).

**Table 2:** Differentially methylated CpGs identified with suggestive significance (unadjusted *p* < 1 × 10^–5^) in the different group comparisons

CpGs	Gene symbol	Delta Beta (Adj)	Delta M-val	P.Value	Adj.P.Val	Chr	Position	Feature	cgi
**ND vs CTRL**									
cg08226295	*SFI1*	0.004	0.50	3.44E-06	0.82	22	31,892,562	5'UTR	Island
cg08003251	*IL22RA2*	− 0.02	− 0.65	3.99E-06	0.82	6	137,469,991	Body	Opensea
cg05296024	*WWOX*	− 0.002	− 0.75	4.56E-06	0.82	16	78,495,308	Body	Opensea
cg21477262	*ETNK1*	0.009	0.56	4.68E-06	0.82	12	22,777,430	TSS1500	Shore
cg23055986	*CEP41*	0.01	0.69	5.72E-06	0.82	7	130,080,444	Body	Shore
cg03068319	*FAM8A1*	0.02	0.51	7.37E-06	0.82	6	17,600,252	TSS1500	Shore
cg23193209	*C4orf50*	− 0.01	− 0.40	7.78E-06	0.82	4	5,972,071	Body	Opensea
cg17839399	*ETNK1*	0.01	0.58	9.14E-06	0.84	12	22,777,409	TSS1500	Shore
**MSA vs CTRL**									
cg22290225	*MEGF11*	− 0.02	− 0.62	1.08E-06	0.80	15	66,319,931	Body	Opensea
**cg15274294**		− **0.07**	− **0.80**	**5.12E-06**	**0.94**	**6**	**14,884,128**	**IGR**	**Opensea**
**cg15644686**	***BCL7B***	− **0.33**	− **2.19**	**9.52E-06**	**0.94**	**7**	**72,972,216**	**TSS1500**	**Island**
**PD vs CTRL**									
cg10828127	*DSCAM*	− 0.004	− 1.40	4.15E-07	0.30	21	41,550,814	Body	Shelf
**cg01380065**	***UBE2F***	**0.10**	**1.33**	**1.25E-06**	**0.46**	**2**	**238,878,242**	**5'UTR**	**Shore**
cg11757352		− 0.01	− 0.64	4.63E-06	0.89	13	30,728,812	IGR	Opensea
cg03068319	*FAM8A1*	0.02	0.56	9.88E-06	0.89	6	17,600,252	TSS1500	Shore
**PSP vs CTRL**									
cg04596067	*MYT1L*	− 0.003	− 0.81	4.34E-06	0.99	2	2,026,609	5'UTR	Opensea
cg04956571	*CBX8*	0.001	0.79	5.28E-06	0.99	17	77,770,388	Body	Shore
cg17839399	*ETNK1*	0.02	0.75	5.37E-06	0.99	12	22,777,409	TSS1500	Shore
cg23193209	*C4orf50*	− 0.02	− 0.52	5.69E-06	0.99	4	5,972,071	Body	Opensea
cg12824502	*MPI*	0.002	0.89	6.85E-06	0.99	15	75,182,590	Body	Island
**cg25358066**	***D2HGDH***	**0.07**	**0.59**	**9.30E-06**	**0.99**	**2**	**242,695,249**	**ExonBnd**	**Opensea**
cg10377240	*FAM179A*	− 0.005	− 0.83	9.41E-06	0.99	2	29,248,433	Body	Opensea
**MSA vs PD**									
**cg05376227**	***FMO6P***	**0.08**	**0.70**	**1.85E-06**	**0.74**	**1**	**171,111,193**	**Body**	**Opensea**
cg07377662	*METRNL*	0.01	0.42	2.24E-06	0.74	17	81,037,199	TSS1500	Island
cg24624576	*SEC63*	0.009	0.65	4.36E-06	0.74	6	108,224,767	Body	Opensea
cg12055395	*DLX6AS*	0.02	1.09	4.86E-06	0.74	7	96,642,605	Body	Shelf
cg07840454	*VN1R1*	0.04	0.57	6.85E-06	0.74	19	57,968,785	TSS1500	Opensea
cg02434357	*SERINC4*	− 0.002	− 0.77	7.41E-06	0.74	15	44,093,254	TSS1500	Shore
cg14932313	*CTSG*	0.03	0.46	8.63E-06	0.74	14	25,043,420	Body	Opensea
**cg20311843**	***OR51A7***	**0.07**	**0.61**	**9.90E-06**	**0.74**	**11**	**4,927,620**	**TSS1500**	**Opensea**
**MSA vs PSP**									
cg04956571	*CBX8*	− 0.001	− 0.80	1.31E-06	0.96	17	77,770,388	Body	Shore
cg22713693	*CUBN*	0.01	0.54	3.17E-06	1.00	10	16,895,852	Body	Opensea
cg02434357	*SERINC4*	− 0.003	− 0.92	7.29E-06	1.00	15	44,093,254	TSS1500	Shore
**PD vs PSP**									
**cg06831571**		**0.12**	**0.90**	**3.82E-06**	**1.00**	**11**	**34,592,196**	**IGR**	**Opensea**
cg00843912	*ZNF180*	− 0.02	− 0.76	4.64E-06	1.00	19	45,004,550	1stExon	Island
cg07366967	*EXD2*	0.03	0.63	7.47E-06	1.00	14	69,675,438	TSS1500	Opensea

*ND* Neurodegenerative parkinsonian disorders, *CTRL* controls, *MSA* multiple system atrophy, *PD* Parkinson’s disease, *PSP* progressive supranuclear palsy. *CpGs* highlighted in bold show absolute delta beta values ≥ 5%

### Specificities of frontal lobe white matter DNA methylation profiles in each of the neurodegenerative parkinsonian disorders

**Fig. 3:**
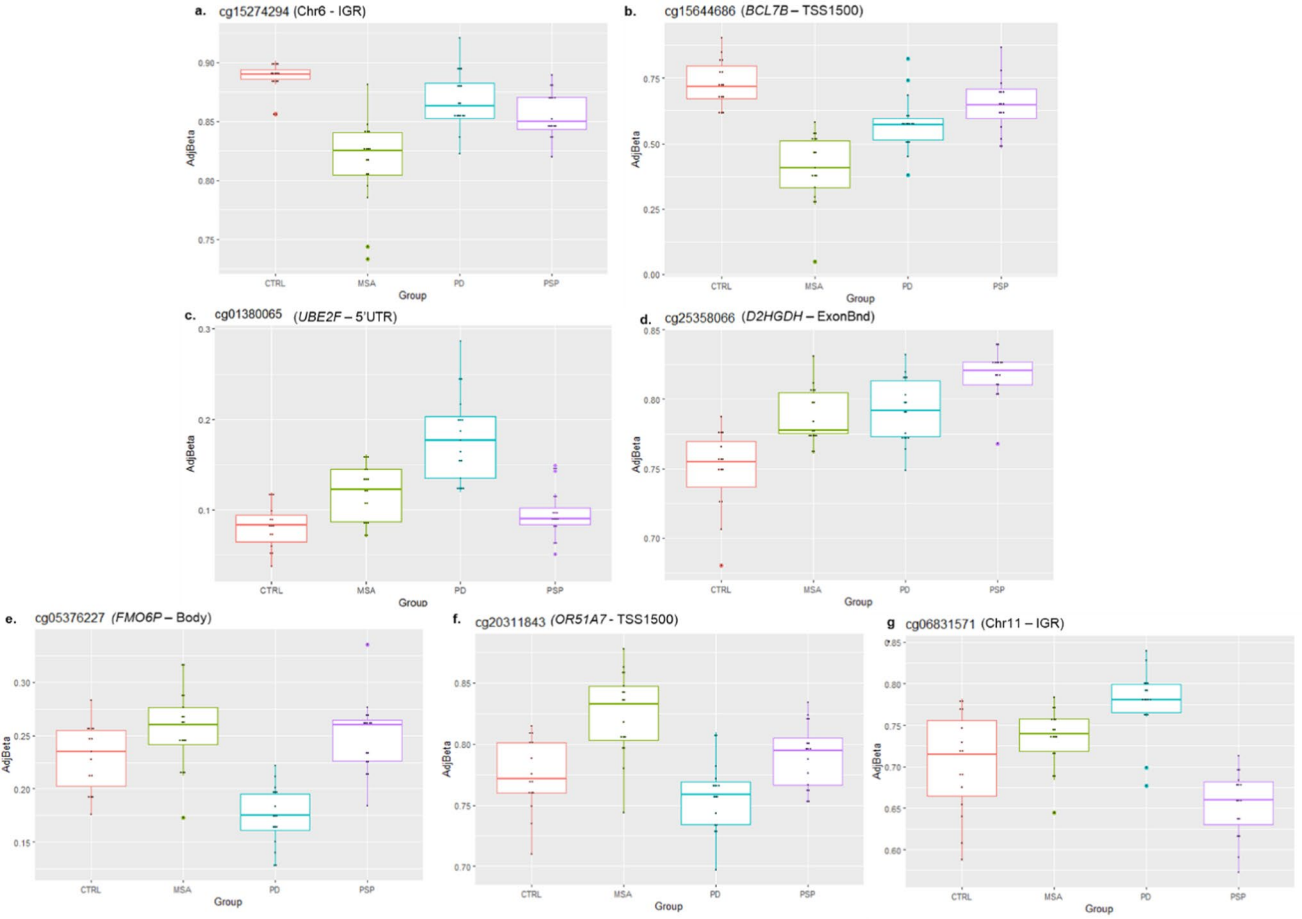
DNA methylation levels for the differentially methylated CpGs in neurodegenerative parkinsonian disorders versus controls showing suggestive significance (unadjusted *p* < 1 × 10^–5^) and effect size (absolute delta beta values) ≥ 5%; **a**, **b** hypomethylation at cg15274294 (Chr 6 – IGR), and cg15644686 (*BCL7B*) in MSA vs controls, **c** hypermethylation at cg01380065 (*UBE2F*) in PD vs controls, **d** hypermethylation at cg25358066 (*D2HGDH*) in PSP vs controls, **e**, **f** hypermethylation at cg05376227 (*FMO6P*), cg20311843 (*OR51A7*) in MSA vs PD and **g** hypermethylation at cg06831571 (Chr11 – IGR) in PD vs PSP. *CTRL* control, *MSA* multiple system atrophy, *PD* Parkinson’s disease, *PSP* progressive supranuclear palsy, *IGR* intergenic region, TSS1500 – 200 -1500 bases upstream of the transcription start site, *ExonBnd* exon boundary

To identify the DNA methylation changes of higher relevance to each disease, we also compared the individual disease groups with controls and identified the top most differentially methylated CpGs (unadjusted *p* < 1 × 10^–5^), which included 3 CpGs in MSA, 4 CpGs in PD, and 7 CpGs in PSP (Supplementary Fig. S2a–c, Table 1). Among these differentially methylated CpGs, only cg15274294 (annotated as intergenic in the Illumina annotations, but found be a novel transcript [ENSG00000234261] associated with the lncRNA class and maps to the novel lincRNA RP11-146I2.1) and cg15644686 (*BCL7B*) in MSA, cg01380065 (*UBE2F*) in PD, and cg25358066 (*D2HGDH*) in PSP showed substantial effect sizes with absolute delta beta values ≥ 5% (Fig. 3); however, the direction of effect for these CpGs remained the same in all three diseases. Notably, there was some overlap of the CpGs identified in the individual disease groups with the CpGs identified in the overall comparison, including CpGs in *FAM8A1*, *C4orf50*, and *ETNK1*. Interestingly, in line with the expected downstream effect of hypomethylation in the promoter region of *BCL7B* (cg15644686) on gene expression levels, a previous study on the transcriptional profiling of cerebellar white matter in MSA reported an average upregulation of *BCL7B* (fold-change = 1.49, adj.P = 2.4 × 10^–2^) in the two cohorts studied [56].

As the majority of the differentially methylated CpGs identified within the disease groups compared to controls showed a similar direction of effect in the three disease groups (Supplementary Fig. S3a–c), we also compared the disease groups against each other to investigate whether there were differential methylation signatures with potential to discriminate between the disease groups (MSA vs PD, MSA vs PSP, and PD vs PSP). Among the top most differentially methylated CpGs in the three parkinsonian disorders (Table 1, Supplementary Fig. S2d–f), only cg05376227 (*FMO6P*), and cg20311843 (*OR51A7*) in MSA vs PD, and cg06831571 (Chr11-intergenic) in PD vs PSP showed effect sizes with absolute delta beta values ≥ 5% (Fig. 3). However, for these CpGs, only the two disease groups being compared showed opposite direction of effects, whereas the other two were always concordant. Interestingly, among the other CpGs in MSA vs PD, one CpG also mapped to the promoter region of gene *VN1R1,* which is a pheromone receptor primarily localized to the olfactory mucosa, similar to *OR51A7*, which is also an olfactory receptor. The overall analysis of the top ranked differentially methylated CpGs (unadjusted *p* < 0.0001) for each comparison also revealed more similarities in DNA methylation patterns between MSA and PD (Supplementary Fig. S3d), both synucleinopathies, compared to that between MSA and PD vs PSP (Supplementary Fig. S3e, f), which is a tauopathy.

### Top differentially methylated positions in parkinsonian disorders are associated with disease traits

**Fig. 4:**
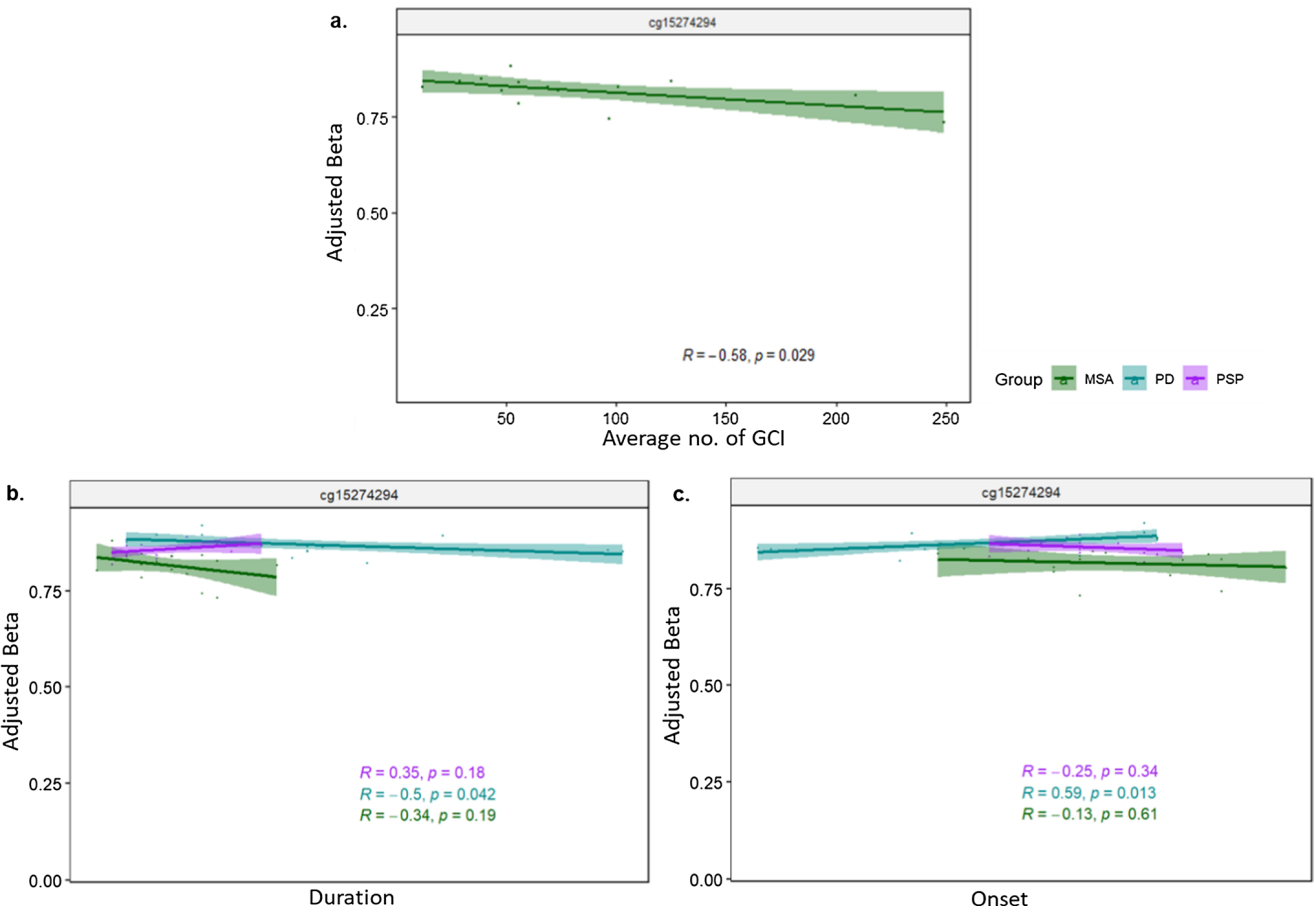
Correlation between differential methylation levels and disease-associated traits for the DMP cg15274294 (Chr 6 – IGR) identified in MSA vs controls. Scatter plot and trend line (Pearson’s correlation) showing correlation between methylation levels and **a** average GCI, **b** disease duration, and **c** disease onset. *MSA* multiple system atrophy (mixed subtype), *PD* Parkinson’s disease, *PSP* progressive supranuclear palsy, *Average no. of  GCI* average number of glial cytoplasmic inclusions

We also explored the top differentially methylated CpGs with effect size ≥ 5% between each disease and controls further to identify associations with disease traits such as the average number of glial cytoplasmic inclusions (GCIs) in the oligodendrocytes in case of MSA, as well as disease onset and duration for all diseases (Fig. 4, Supplementary fig. S4). We found that methylation levels at the intergenic/lincRNA cg15274294 in MSA were inversely associated with the mean number of GCIs in the frontal lobe (*R* = − 0.58, *p* = 0.029) (Fig. 4a), and the same direction of effect was observed for disease duration (*R* = − 0.34, n.s.); in PD, lower methylation levels in this CpG also showed significant correlations with earlier onset of disease (*R* = 0.59, *p* = 0.013), but longer disease duration (*R* = − 0.50, *p* = 0.042) (Fig. 4b,c). It is of note that this CpG (cg15274294), although annotated as intergenic in the Illumina manifest, maps to a novel lincRNA transcript [ENSG00000234261], which in the healthy brain exhibits higher expression levels within the basal ganglia regions that are relevant for both MSA and PD (Supplementary Fig. S5). Overall, these findings suggest that the methylation status at this site is related with disease progression in synucleinopathies. No other significant correlations were found for the remaining CpGs resulting from the comparisons between disease and controls (Supplementary Fig. S4).

Among the CpGs showing the strongest effects in the comparisons between disease groups, a significant negative correlation was also observed between methylation levels in cg05376227 (*FMO6P*) and avgGCI in MSA (*R* = − 0.59, *p* = 0.026) (Supplementary Fig. S6a); a significant negative correlation was also observed between cg20311843 (*OR51A7*) and disease onset in PSP (*R* = − 0.59, *p* = 0.015) (Supplementary Fig. S6b); no significant correlations were observed with disease duration (Supplementary Fig. S6c).

### WGCNA identifies shared and disease-specific DNA co-methylation modules

**Fig. 5:**
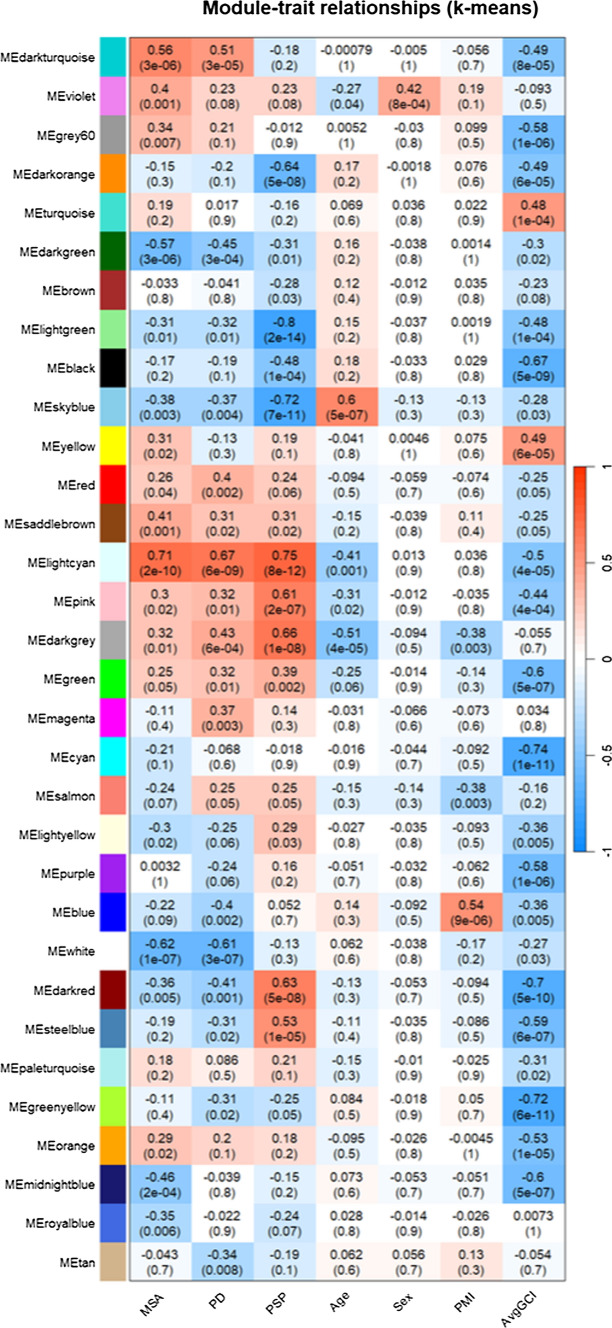
Module-trait correlations for the co-methylation networks. Rows represent co-methylation module eigengenes and their colors; columns represent the correlation (and p-values) of the methylation levels of CpGs in each module with the disease status and clinical/pathological traits. Color scale at the right indicates the strength of the correlation (darker cells depict stronger correlations, with blue representing negative and red representing positive correlations)

We further performed co-methylation analysis using WGCNA to identify clusters of highly correlated CpGs (modules) (Supplementary Fig. S7). A total of 32 co-methylation modules were identified, 15 of which were significantly associated with the status of at least one disease group (*p* ≤ 0.0015, 0.05/32 modules) (Fig. 5, Supplementary Fig. S8a). Among these, the lightcyan module was positively associated with all three disease groups [MSA (*R* = 0.71, *p* = 2 × 10^–10^), PD (*R* = 0.67, *p* = 6 × 10^–9^), and PSP (*R* = 0.75, *p* = 8 × 10^–12^)] (Fig. 5), whereas the darkgray module showed a positive correlation in both PD and PSP [PD (*R* = 0.43, *p* = 6 × 10^–4^); PSP (*R* = 0.66, *p* = 1 × 10^–8^)] and to a lower extent in MSA (*R* = 0.32, *p* = 0.01). Modules significantly associated only with α-synucleinopathies included darkturquoise [MSA (*R* = 0.56, *p* = 3 × 10^–6^); PD (*R* = 0.51, *p* = 3 × 10^–5^)], darkgreen [MSA (*R* = − 0.57, *p* = 3 × 10^–6^); PD (*R* = − 0.45, *p* = 3 × 10^–4^)], and white [MSA (*R* = − 0.62, *p* = 1 × 10^–7^); PD (*R* = − 0.61, *p* = 3 × 10^–7^)] (Fig. 5, Supplementary Fig. S8b). As with the differential methylation analysis, both synucleinopathies had more similarities among them than with PSP, with concordant direction of effects in all disease-associated modules for MSA and PD (Fig. 5).

Modules significantly associated with MSA only included violet (*R* = 0.4, *p* = 0.001), saddlebrown (*R* = 0.41, *p* = 0.001) and midnightblue [*R* = − 0.46, *p* = 2 × 10^–4^ (Fig. 5, Supplementary Figs. S8a and S9a)]. The PSP-associated darkred module showed an inverse association with both α-synucleinopathies [MSA (*R* = − 0.36, *p* = 0.005), PD (*R* = − 0.41, *p* = 0.001); PSP (*R* = 0.63, *p* = 5 × 10^–8^)]. Similarly, the PSP-associated steelblue module (*R* = 0.53, *p* = 1 × 10^–5^) showed non-significant inverse correlations with both α-synucleinopathies [MSA (*R* = − 0.19, n.s.), PD (*R* = − 0.31, *p* = n.s.)] (Fig. 5, Supplementary Figs. S8a and S9c). Other modules significantly associated with PSP, but showing non-significant associations in the same direction with MSA and PD included the pink module (*R* = 0.61, *p* = 2 × 10^–7^), light green (*R* = − 0.8, *p* = 2 × 10^–14^), sky blue (*R* = − 0.72, *p* = 7 × 10^–11^), darkorange (*R* = − 0.64, *p* = 5 × 10^–8^), and black modules (*R* = − 0.48, *p* = 1 × 10^–4^) (Fig. 5, Supplementary Figs. S8a and S9c). No PD-specific modules were identified.

We also investigated whether the disease-associated modules were associated with clinical and pathological traits, such as age of disease onset and disease duration, and the average number of GCIs (avgGCI) in MSA. The darkred module positively associated with PSP also showed negative correlation with disease duration in PSP, suggesting higher methylation levels in the CpG sites within this module could contribute toward a faster progression and shorter disease duration (Supplementary Figs. S8). Among the modules significantly associated with the MSA status, the darkturquoise, the lightcyan, and the midnightblue also showed negative correlations with avgGCI (Fig. 5), supporting a role of DNA methylation in the progression of the MSA pathology.

We further explored each of the modules significantly associated with one or more disease groups and investigated module memberships to identify the most interconnected genes within those modules (i.e., intramodular hub genes). Hub gene analysis highlighted dysregulation in several genes previously implicated in neurodegeneration as well as in parkinsonian disorders (Supplementary Table S2). Interestingly, the pyroptotic gene *DFNA5*, involved in a specialized and pro-inflammatory form of programmed cell death [58], was identified as the top hub gene in the lightcyan module commonly dysregulated in all three diseases. Hub genes of the α-synucleinopathy-associated modules included *RBP4*,* C1orf70 (TMEM240)*, and *SCARF2*, which have been previously implicated in PD [73]. Additionally, CpGs with the higher module membership within a given disease-associated module (e.g., lightcyan module) were often associated with higher gene significance, suggesting those are biologically more relevant to disease (Supplementary Fig. S9).

### Disease-associated co-methylation modules enriched for oligodendrocytic gene signatures display enrichment of distinct molecular pathways involved in neurodegeneration

**Fig. 6:**
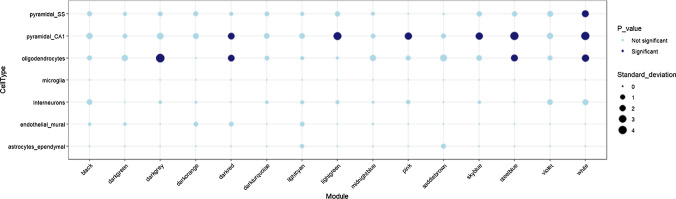
Cell type enrichment for the WGCNA modules associated with one or more disease groups. Enrichment for the different brain cell types performed using the EWCE package and associated single-cell transcriptomic data which uses mouse to human homologs of genes associated with various cell types; dark blue circles represent significantly enriched cell types with adjusted *p* < 0.05 after Bonferroni corrections; the size of the circles represents the number of standard deviations (SD) from the mean

As we analyzed DNA methylation in the white matter, we performed cell type enrichment analysis to identify modules significantly enriched for specific glial cells and further understand their contribution disease-related processes. The darkgray, darkred, steelblue, and white modules showed a significant enrichment for oligodendrocytic gene signatures (Fig. 6). When assessing enrichment for specific subpopulations within the oligodendrocyte lineage, the midnightblue module was specifically enriched for the sub-cell type Oligo1, which is thought to correspond to oligodendrocytes undergoing differentiation (Supplementary Fig. S10) [78]. The steelblue and white modules showed significant enrichments for Oligo2 and Oligo6, which represent pre-myelinating and terminally differentiated post-myelinating oligodendrocytes, respectively [78]. No significant enrichment was observed for astroglial and microglial cell types. Neuronal proportion estimates within the dataset were negligible, and therefore, any enrichment for neuronal markers was interpreted as related with neuronal signatures being silenced in the white matter.

**Fig. 7:**
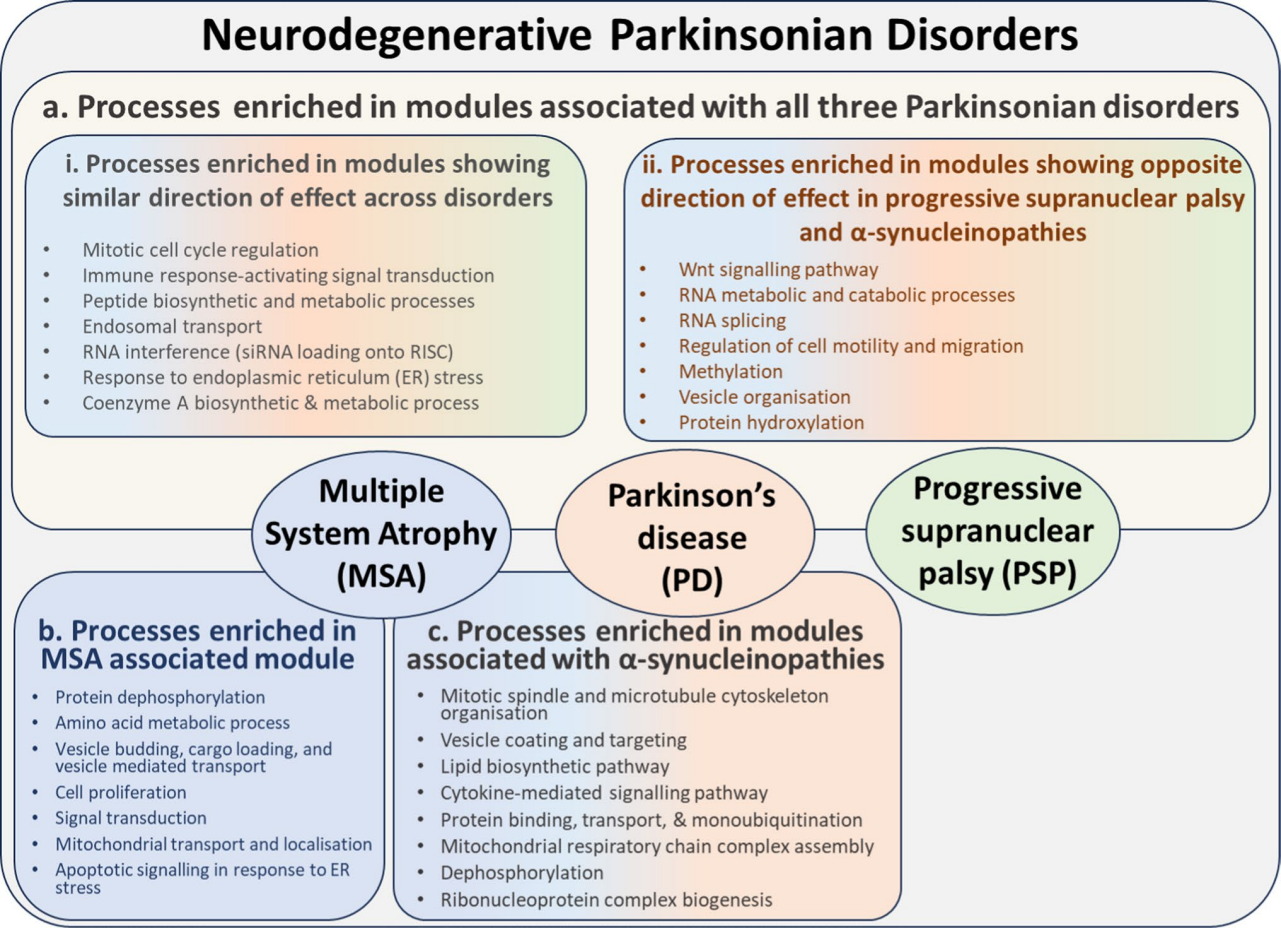
Summary of enriched pathways and processes obtained from the frontal lobe specific functional network analysis on the parkinsonian disorders-associated oligodendrocyte-enriched co-methylation modules. **a** Processes that were commonly enriched in all three neurodegenerative Parkinsonian disorders. These were further classified based on processes enriched in (i) modules that showed similar direction of effect across the three disorders and (ii) modules that showed opposite direction of effect in PSP and α-synucleinopathies, **b** processes that were specifically enriched within the modules associated with MSA, **c** processes that were commonly enriched within the modules associated with α-synucleinopathies

As our main objective was to elucidate the contribution of DNA methylation perturbation to the parkinsonian disorders in tissues enriched for specific glial cell types, we focused our attention on further exploring modules specifically associated with oligodendrocyte signatures. We therefore performed gene ontology enrichment and functional network analysis specific for the frontal lobe for genes in the oligodendrocyte-enriched co-methylation modules to understand their functional significance. The darkgray module, which was significantly associated with PD and PSP, and to a certain extent with MSA, showed submodules enriched for processes such as RNA interference (M1), immune response-activating signal transduction (M4), regulation of exosomal secretion, response to endoplasmic reticulum (ER) stress, regulation of mitochondrial translation (M5), and endosomal transport (M6) (Fig. 7, Supplementary Fig. S11a, Supplementary Table S3). The white module significantly associated specifically with the α-synucleinopathies (MSA and PD) showed its largest submodule (M3) to be enriched for processes such as regulation of Wnt signaling pathway and cell–cell signaling by Wnt (Fig. 7, Supplementary Fig. S11b, Supplementary Table S3). Interestingly, this submodule had PARKIN (encoded by *PARKN*, a causal gene in familial forms of PD [14]) as its hub, and also contained our top MSA differentially methylated CpG in *BCL7B* (cg15644686). Processes specifically enriched in α-synucleinopathies included the lipid biosynthetic pathway, vesicle coating and targeting, among others. The darkred and the steelblue modules showed negative associations with α-synucleinopathies and a positive association with PSP. The largest submodule in darkred (M8) was enriched for processes involved with histone methylation, cell migration, and Wnt signaling pathway, again with PARKIN as its hub (Fig. 7, Supplementary Fig. S11c, Supplementary Table S3). The steelblue module was enriched for processes such as protein localization to nucleoplasm (M2), RNA splicing and translation (M1), and antigen processing and presentation (M3) (Fig. 6, Supplementary Fig. S11d, Supplementary Table S3). The midnightblue module was the only module exclusively associated with MSA and showed an enrichment for negative regulation of Wnt signaling pathway, cellular response to lipid, and regulation of SMAD protein signal transduction in the M4 submodule and processes such as regulation of protein dephosphorylation, protein targeting and localization to mitochondrion, and apoptotic signaling in response to ER stress (Fig. 7, Supplementary Fig. S11e, Supplementary Table S3).

### Disease-associated co-methylation modules are preserved to various degrees in other brain regions and tissue types in MSA, Lewy body diseases (LBD), and PSP, and display overlaps in dysregulated processes

**Fig. 8:**
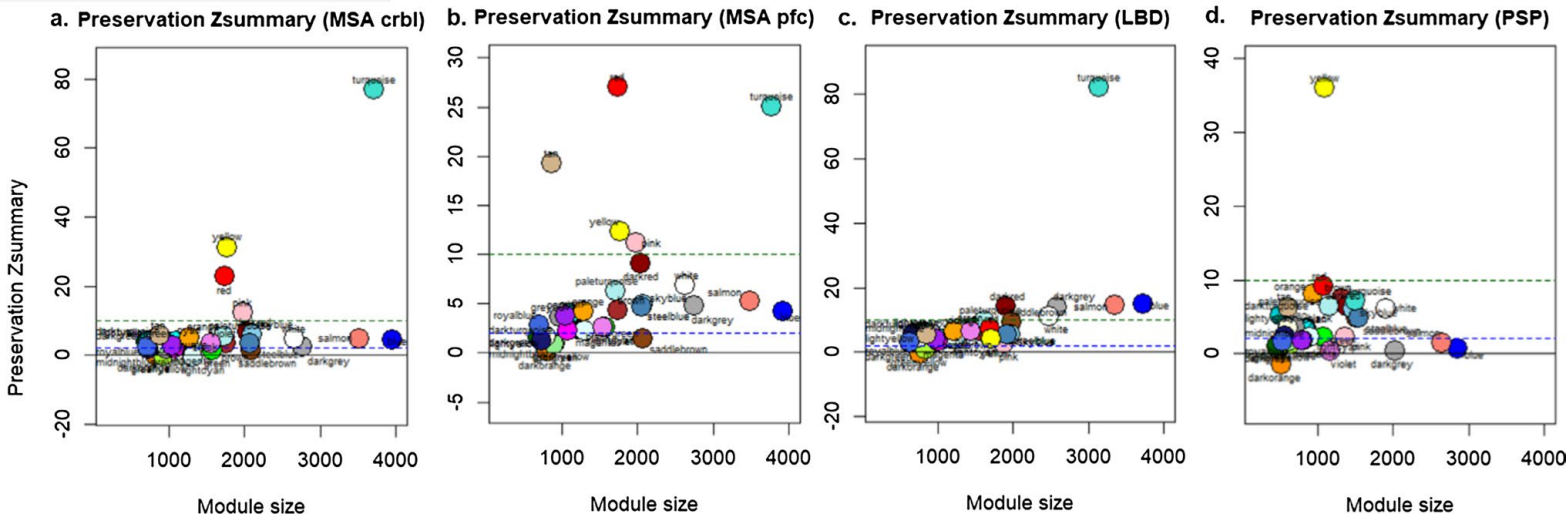
Preservation analysis for the co-methylation modules in the frontal lobe white matter dataset for MSA, PD, and PSP in other brain regions and tissue types. Preservation Z summaries of the co-methylated modules in **a** MSA cerebellar white matter dataset, **b** MSA prefrontal cortex gray and white matter dataset, **c** Lewy body disease (LBD) frontal cortex gray matter dataset, and **d** PSP prefrontal lobe gray and white matter dataset. Y-axis represents the preservation Z-summary with modules above the green dashed line (Z-summary > 10) predicted to be highly preserved, those between the blue and green dashed lines (Z-summary 2–10) predicted to be moderately preserved, and modules below the blue line (Z-summary < 2) are not preserved. *MSA* multiple system atrophy, *PD* Parkinson’s disease, *PSP* progressive supranuclear palsy

To assess whether the parkinsonian disorder-associated white matter co-methylation modules have a broader disease relevance beyond frontal lobe white matter, we performed preservation analysis using multiple previously available datasets from other brain regions, tissue types, and diseases. These included DNA methylation profiles of MSA cerebellar white matter, MSA prefrontal cortex gray and white matter mixed tissue, LBD frontal cortex gray matter, and PSP prefrontal lobe gray and white matter mixed tissue datasets. Most disease-associated modules displayed moderate to high preservation (Z-summary 2–10 and > 10, respectively) within these datasets, such as the oligodendrocyte associated midnightblue and white modules in the cerebellar white matter dataset (Fig. 8a), the white module in MSA prefrontal cortex gray and white matter (Fig. 8b), modules darkgray, darkred, and white in the LBD dataset (Fig. 8c), and modules darkred and steelblue in the PSP dataset (Fig. 8d).

We further performed WGCNA on the abovementioned additional datasets to identify functional similarities between disease-associated modules identified in our white matter cross-disease dataset and those identified in the other datasets comprising different brain regions and tissue types. Several overlapping disrupted processes and pathways were identified from the functional enrichment and network analyses of these datasets (Table S4). Modules associated with MSA in the cerebellar white matter dataset and MSA in our dataset demonstrated shared enrichment for processes such as protein phosphorylation, cell migration, and cell motility. In case of the MSA prefrontal cortex gray and white matter mixed tissue dataset, common enriched pathways included protein dephosphorylation, regulation of peroxisome organization, and intracellular protein transport, among others. Modules associated with LBD frontal cortex gray matter and PD in our dataset exhibited shared processes such as transmembrane receptor protein tyrosine kinase signaling pathway. Enriched processes common between modules associated with PSP prefrontal cortex gray and white matter and PSP in our dataset included regulation of mRNA metabolic process, DNA metabolic process, and chromosome organization, response to endoplasmic reticulum stress, histone methylation, endosomal transport, and positive regulation of TOR signaling (Table S4).

## Discussion

We performed a cross-comparative analysis of DNA methylation changes in the frontal lobe white matter of individuals with MSA, PD, and PSP to identify shared and disease-specific molecular signatures in the white matter. Despite the variable extent of white matter involvement across these three parkinsonian disorders [16], a comprehensive analysis revealed substantial commonalities in DNA methylation alterations, with a majority of the differentially methylated CpGs displaying a similar direction of effect across diseases, albeit with varying effect sizes. This shared DNA methylation architecture suggests that the presence of similar pathogenic mechanisms and cellular responses in MSA, PD, and PSP extends to (cell types within) the frontal lobe white matter. Among the three parkinsonian disorders, trends pointed toward greater similarities in MSA and PD compared to MSA and PSP or PD and PSP both in terms of the differential methylation analysis as well as with the co-methylation networks, where a higher number of co-methylated disease-associated modules and higher similarities in the direction of effect between MSA and PD were observed compared to those observed between MSA and PSP or PD and PSP. Together, these observations suggest that the synucleinopathies might share more commonalities in terms of DNA methylation, despite differences in the cell types where α-synuclein inclusions primarily occur. Therefore, despite the more extensive white matter involvement in MSA and PSP relative to PD [51], the aggregated protein type (α-synuclein vs. tau) might introduce a greater degree of difference in the underlying molecular processes, as reflected in our overall DNA methylation analysis.

The top differentially methylated CpGs identified with suggestive significance commonly altered in MSA, PD, and PSP, mapped to several genes with prior associations with neurological conditions. For instance, *IL22RA2*, is a multiple sclerosis risk gene [9] and has been shown to play a role in oligodendrocytic apoptosis [79]. *WWOX*, an AD risk gene also implicated in PD and multiple sclerosis [2, 29], has been shown to be responsible for Tau hyperphosphorylation resulting in aggregation of Tau into neurofibrillary tangles (NFTs), in addition to possessing pro-apoptotic properties, and its loss-of-function has been shown to result in the activation of a protein aggregation cascade [34]. *ETNK1* plays crucial roles in the folding and activity of several membrane proteins, initiation of autophagy, maintaining optimal mitochondrial respiratory activity and ubiquinone function [23, 53]. The observed increased methylation of two CpGs in the promoter region of *ETNK1* suggests a dysregulation/repression of *ETNK1*, potentially resulting in protein misfolding and aggregation due to abnormal protein degradation (impaired autophagy) that is characteristic of these diseases. *FAM8A1* is involved in ubiquitin-dependent endoplasmic reticulum-associated degradation of proteins with roles in AD pathogenesis, and a differentially methylated CpG mapping to *FAM8A1* was also the most significantly associated with AD in a previous study [69]. Additionally, *DFNA5*, the hub gene identified in the WGCNA module commonly associated with the three parkinsonian disorders, is a pyroptotic gene reported to induce programmed cell death through mitochondria and MAPK-related pathways [68] and mediates mitochondrial damage in axons and neurodegeneration [50]. Differential methylation of these loci commonly identified across the parkinsonian disorders suggests commonalities in terms of pathways related to autophagy, mitophagy, apoptosis, and protein degradation pathways in all these diseases. Although all these pathways have previously been linked to these diseases, our findings highlight a role of white matter DNA methylation changes in the dysregulation of such pathways.

Among the CpGs identified in the individual comparisons of each disease group with controls, hypomethylation in the promoter region at cg15644686 mapping to *BCL7B* was observed in MSA, with concordant transcriptional upregulation being reported in the MSA cerebellar white matter [56]. BCL7B (BAF chromatin remodeling complex subunit BCL7B) is a negative regulator of Wnt signaling and a pro-apoptotic factor, and a deficiency in BCL7B reportedly enhances oligodendrogenesis [30, 67, 76]. This may suggest that increased levels of BCL7B in MSA might hinder oligodendrogenesis. This, in conjunction with the gliosis and demyelination observed in MSA, could exacerbate disease pathology, as indicated by the inverse correlation between methylation levels and disease duration and the higher mean number of GCIs in MSA, as we observed in our study. The CpG cg01380065 showed hypermethylation in PD compared to controls. This CpG maps to *UBE2F*, which has been shown to be involved in neddylation, which is a post-translational modification essential for regulating the clearance of misfolded proteins [27]. The CpG cg25358066 (ExonBnd), found to be hypermethylated in with the strongest effect in PSP, mapped to *D2HGDH* (D-2-hydroxyglutarate dehydrogenase), a mitochondrial enzyme belonging to the FAD-binding oxidoreductase/transferase type 4 family and an overexpression of D2HGDH has been demonstrated to inhibit ferroptosis [73]. Therefore, differential methylation at this site might contribute toward dysregulation in the ferroptosis pathway in PSP [75].

Our findings revealed that the extent of disease specificity in terms of differential methylation in the frontal lobe white matter between these three diseases is limited. Although a few differentially methylated CpGs that showed opposite direction of effect in one disease compared to the other were identified, such as *FMO6P* and *OR51A7*, in MSA compared to PD, in most cases, dysregulation was still observed with the CpG in all three disease groups. However, the presence of multiple differentially methylated CpGs within the olfactory receptor genes (*OR51A7, VN1R1*) in the MSA vs PD comparison group suggests that DNA methylation changes in olfactory genes and pathways related to olfaction could be a factor discriminating PD from MSA and PSP. This is further in line with previous reports of absence of a history of hyposmia or anosmia in patients with MSA and that hyposmia in PSP suggests the presence of additional Lewy body pathology [35, 40]. Other, notable hub genes specifically identified in modules correlating with α-synucleinopathies included *C1orf70*, which has been implicated to play a role in spinocerebellar ataxia, and *SCARF2,* which maps to the 22q11 deletion region previously associated with increased PD risk, suggesting that dysregulation of these genes might also be involved in the MSA pathogenesis [45].

Co-methylation modules enriched for oligodendrocytic genes included some modules commonly associated with all three parkinsonian disorders, and some that were associated with synucleinopathies only, in addition to some disease-specific modules. The oligodendrocyte-enriched darkgray module, significantly positively associated with PD, PSP and to a certain extent MSA, showed functional enrichment of processes, such as RNA interference (RNAi), signal transduction, ER stress, mitochondrial translation, and endosomal transport, suggesting a common involvement of these molecular pathways in all three parkinsonian disorders. Mechanisms relating to RNAi have already been reported for several neurodegenerative diseases including in PD and therapeutic models of RNAi are being extensively studied in animal models of HD, AD, and PD [24]. Intra- or inter-cellular signaling mechanisms have also been described to be involved in the pathogenesis of neurodegenerative diseases with effectors and/or components of the signal transduction pathways playing important roles in progression, and possibly in the initiation, of these diseases [72]. Mechanisms relating to ER stress, mitochondrial functions and endosomal transport have also been extensively reported in neurodegenerative diseases including PD, with PARK17 playing a role in the retrotransfer of proteins from endosomes in the pre-lysosomal compartment network to the trans-Golgi network, and PARK9 and ATP13A2 coding for endo-/lysosomal-related proteins, HTRA2 (PARK13) being crucial to maintaining normal mitochondrial function and ERS-coupled apoptotic cell death being implicated in neurodegeneration [17, 65].

The oligodendrocyte-enriched white module, which was significantly associated commonly in the synucleinopathies (MSA and PD), showed enrichment of processes relating to the Wnt signaling pathway. Wnt signaling pathway has previously been shown to play an important role in PD pathogenesis, with dysfunction in PARKIN, leading to the accumulation of β-catenin and resulting in the upregulation of canonical Wnt signaling. Interestingly, PARKIN was the most important hub in the M3 functional module within the white module and also contained our MSA top hit in *BCL7B*, which has been shown to play a role in the Wnt signaling pathway by negatively regulating the expression of Wnt signaling components CTNNB1 and HMGA1 [67]. Put together, these findings indicate DNA methylation dysregulation in Wnt signaling pathways to be common in both MSA and PD even in the white matter. Moreover, white matter damage has been found to precede gray matter atrophy in both MSA and PD [1, 12, 19]. Wnt signaling pathways play important roles in oligodendrogenesis, oligodendrocyte differentiation, and myelination, and DNA methylation alterations dysregulating the Wnt signaling pathway might be one of the factors responsible for preventing remyelination through the mobilization of OPCs following death of oligodendrocytes or myelin damage due to disease [26, 59].

Co-methylation modules enriched for oligodendrocytes significantly associated with PSP included the darkred and steelblue modules, both of which also showed inverse associations with synucleinopathies. Both these modules showed enrichment in processes related to RNA splicing, mRNA and peptide metabolic processes. The darkred module was also enriched for histone methylation. The inverse correlation observed between the synucleinopathies and tauopathy could be attributed to differences in these molecular processes or different molecular players within these processes driving the pathogenesis. Additionally, the darkred module also showed enrichment for the Wnt signaling pathway, which has also been reported in a previous DNA methylation study conducted in PSP forebrains [74], suggesting that DNA methylation alterations within this pathway is a common factor in white matter tissues across MSA, PD, and PSP. The MSA associated midnightblue module, in addition to being enriched for the commonly identified Wnt signaling and apoptotic processes, also showed enrichment in ER pathways, such as COPII-coated vesicle budding and cargo loading into COPII-vesicle, protein dephosphorylation, and cytokine-mediated signaling pathway suggesting additional roles of these pathways in MSA pathogenesis.

As any other DNA methylomic study, our study also has several limitations. The Illumina EPIC array, while comprehensive, might miss methylation changes not covered by the predefined methylation sites included in the array. Although the analysis of frontal lobe white matter DNA methylation profiles revealed several commonalities in MSA, PD, and PSP, brain regions and tissue types primarily affected in the three diseases vary. DNA methylation differences in the primary affected region in each disease, such as substantia nigra/basal ganglia in PD, striatum, substantia nigra and cerebellum in MSA, and subcortical and cortical regions in PSP, could not be captured in this study as we focused on the frontal lobe white matter. However, we chose the frontal lobe as it shows moderate to high pathology at the end-stage in all three diseases, to be able to perform a cross-disease comparison. Therefore, further studies examining the extent of dysregulation of the identified DNA methylation alterations in other brain regions might provide additional insights into disease-specific patterns. Furthermore, we focused on the frontal lobe white matter, which is naturally highly enriched for oligodendrocytes and should reflect DNA methylation changes mostly specific to this cell type. However, the different cellular populations, including neurons, oligodendrocytes, and other glial cells, within other tissue types could not be captured and further studies are warranted to identify other brain region and cell type-specific changes. Moreover, as our study uses post-mortem brain tissues, we cannot identify early changes in DNA methylation, and cannot distinguish the causative DNA methylation alterations from the reactive changes. In addition, we cannot completely rule out the effects of age, as the MSA and PSP cases were generally younger than the controls. The potential impact of medications like levodopa on DNA methylation also cannot be ruled out. Our modest sample sizes per group only made it possible to identify differential DNA methylation alterations at nominal significance. However, we compensated this limitation by employing more powerful system biology approaches such as co-methylation networks to further complement and strengthen our findings. Furthermore, we used additional datasets, which further validated shared disturbed processes across parkinsonian disorders in different brain regions and/or tissue types. While we identified DNA methylation alterations disrupting interesting pathways, most of which linked to these diseases at other levels, bioinformatic functional enrichment approaches rely on existing pathway databases and annotations, which may not be comprehensive for all biological contexts, and functional validation is warranted in follow-up studies.

In conclusion, our study provides the first evidence in the white matter of three different parkinsonian disorders that point to common DNA methylation alterations and shared relevant pathogenic mechanisms in the three diseases. As imaging studies report that white matter changes often happen early in neurodegenerative diseases, including the ones studied here, it is key to understand the molecular mechanisms underpinning such changes. Our study reveals the overall presence of more similarities than differences in MSA, PD, and PSP frontal lobe white matter in terms of DNA methylation architecture, with differences between diseases primarily lying in the effect sizes of the alterations. While this study identifies shared mechanisms that provide valuable insights into the DNA methylation changes in parkinsonian disorders, further studies involving larger sample sizes and multiple regions and tissue/cell types of the brain are warranted for the identification of disease-specific methylation changes in these diseases. The integration of DNA methylation data with other omics datasets, such as transcriptomics, proteomics, and metabolomics, in future studies should provide a more comprehensive picture of the molecular processes and pathways involved in these disorders. Nevertheless, our study identifies several candidate loci and pathways that display shared DNA methylation dysregulation in the frontal lobe white matter in all three Parkinsonian disorders that can be further explored as potential therapeutic targets and highlights common pathogenic mechanisms between the diseases, which are indicative of converging molecular pathways that contribute to neurodegeneration in MSA, PD, and PSP.

### Supplementary Information

Below is the link to the electronic supplementary material.

Supplementary file1 (PDF 2882 KB)

## Data Availability

Raw methylation data for the MSA prefrontal cortex, LBD, and PSP prefrontal lobe datasets are available in NCBI GEO database (https://www.ncbi.nlm.nih.gov/geo), and can be accessed via accession numbers GSE143157, GSE203332, GSE197305, and GSE75704. Additional data is available in supplementary materials and from the corresponding author upon reasonable request.
