# Supplementary material for: DNA methylation patterns in the frontal lobe white matter of multiple system atrophy, Parkinson’s disease, and progressive supranuclear palsy: a cross-comparative investigation
Source: Acta Neuropathol. 2024 Jul 12;148(1):4. doi: 10.1007/s00401-024-02764-4 (PMC11245434; doi:10.1007/s00401-024-02764-4)
Supplement: Supplementary file 1 — Supplementary file1 (PDF 2882 KB) [file 401_2024_2764_MOESM1_ESM.pdf]

DNA methylation patterns in the frontal lobe white matter of multiple system atrophy, Parkinson's disease, and progressive supranuclear palsy: A cross-comparative investigation

Megha Murthy[1,2], Katherine Fodder[1,3], Yasuo Miki[4], Naiomi Rambarack[1,2], Eduardo De Pablo Fernandez[1,2,5], Lasse Pihlstrøm[6], Jonathan Mill[7], Thomas T Warner[1,2,5], Tammaryn Lashley[3], Conceição Bettencourt[1,3,*]

1. Queen Square Brain Bank for Neurological Disorders, UCL Queen Square Institute of Neurology, London, UK.
2. Department of Clinical and Movement Neurosciences, UCL Queen Square Institute of Neurology, London, UK.
3. Department of Neurodegenerative Disease, UCL Queen Square Institute of Neurology, London, UK.
4. Department of Neuropathology, Institute of Brain Science, Hirosaki University Graduate School of Medicine, Hirosaki, Japan.
5. Reta Lila Weston Institute, UCL Queen Square Institute of Neurology, London, UK.
6. Department of Neurology, Oslo University Hospital, Oslo, Norway
7. Department of Clinical and Biomedical Sciences, Faculty of Health and Life Sciences, University of Exeter, Exeter, UK.

***Corresponding Author:**

Conceição Bettencourt, PhD

Department of Neurodegenerative Disease

UCL Queen Square Institute of Neurology

1 Wakefield Street

London WC1N 1PJ

United Kingdom

Email c.bettencourt@ucl.ac.uk

Supplementary Figures

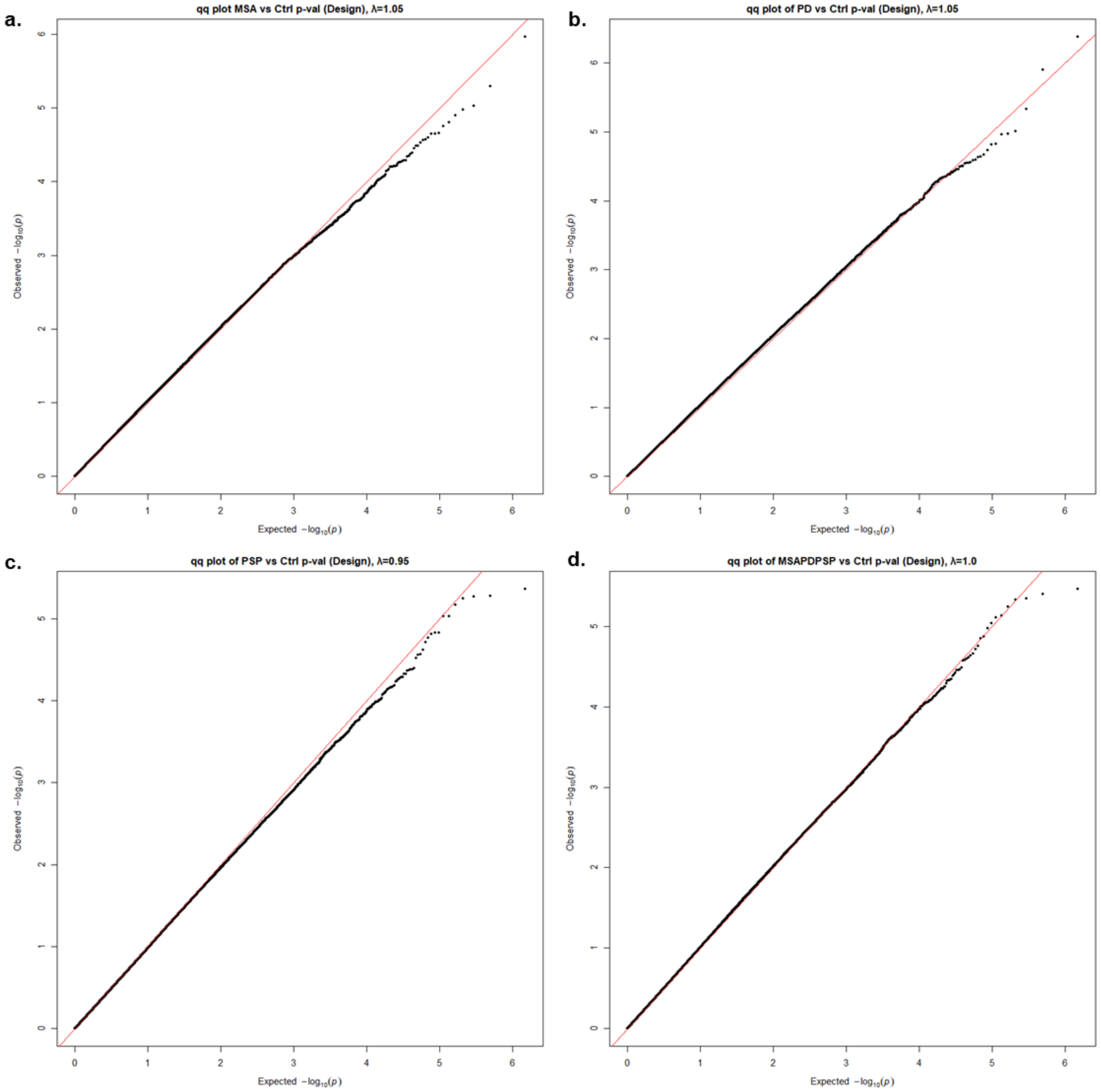

Supplementary fig. S1 Quantile-quantile (Q-Q) plots for the disease group specific case-control EWAS (a) Q-Q plot for MSA shows an estimated inflation factor (λ) of 1.05, (b) Q-Q plot for PD shows an estimated inflation factor (λ) of 1.05, (c) Q-Q plot for PSP shows an estimated inflation factor (λ) of 0.95, (d) Q-Q plot for all disease groups shows an estimated inflation factor (λ) of 1.0. CTRL – controls, MSA – multiple system atrophy, PD – Parkinson's disease, PSP – progressive supranuclear palsy

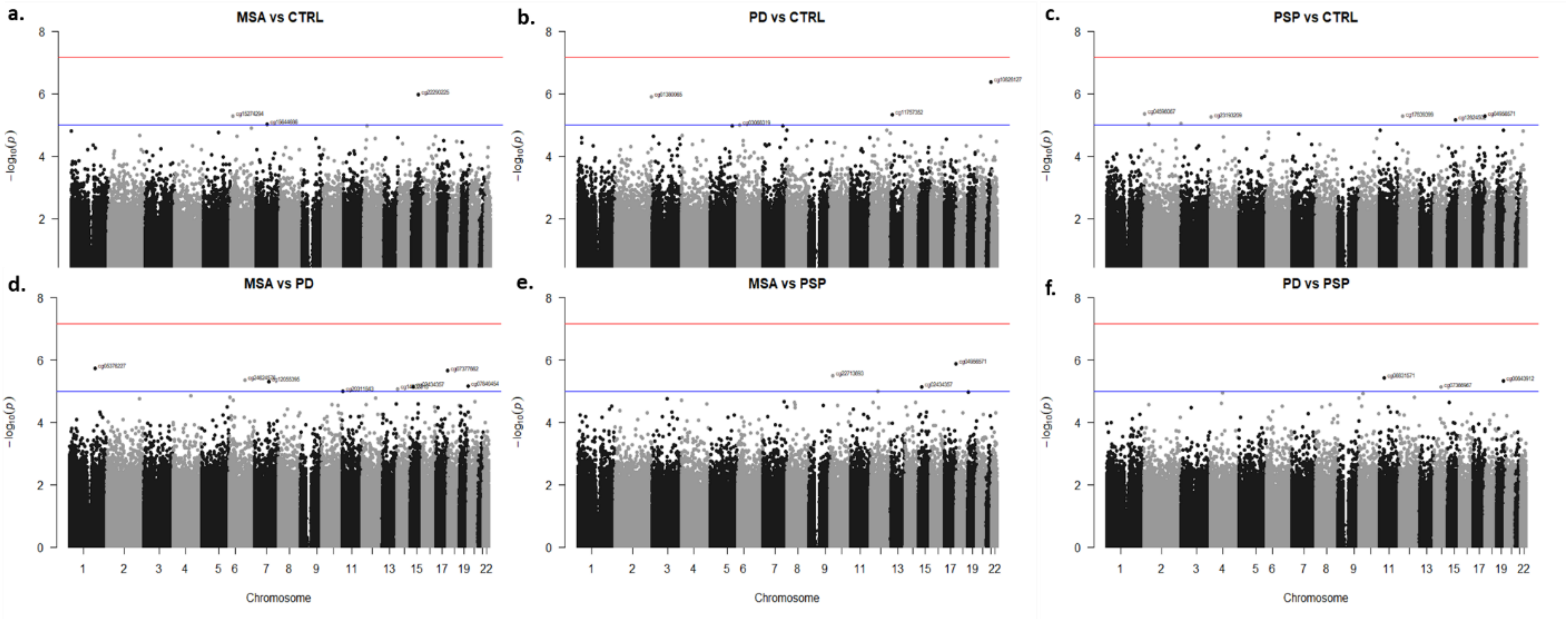

Supplementary fig. S2 Manhattan plots showing the p-value distributions of the differentially methylated CpGs for the different comparisons, (a.) MSA vs CTRL, (b.) PD vs CTRL, (c.) PSP vs CTRL, (d.) MSA vs PD, (e.) MSA vs PSP, (f.) PD vs PSP. The red line indicates genome-wide significance threshold based on Bonferroni-corrected p-values ($p = 6.8 \times 10^{-8}$), and the blue line indicates a less stringent suggestive significance threshold of $p = 1 \times 10^{-5}$. CTRL – controls, MSA – multiple system atrophy, PD – Parkinson's disease, PSP – progressive supranuclear palsy

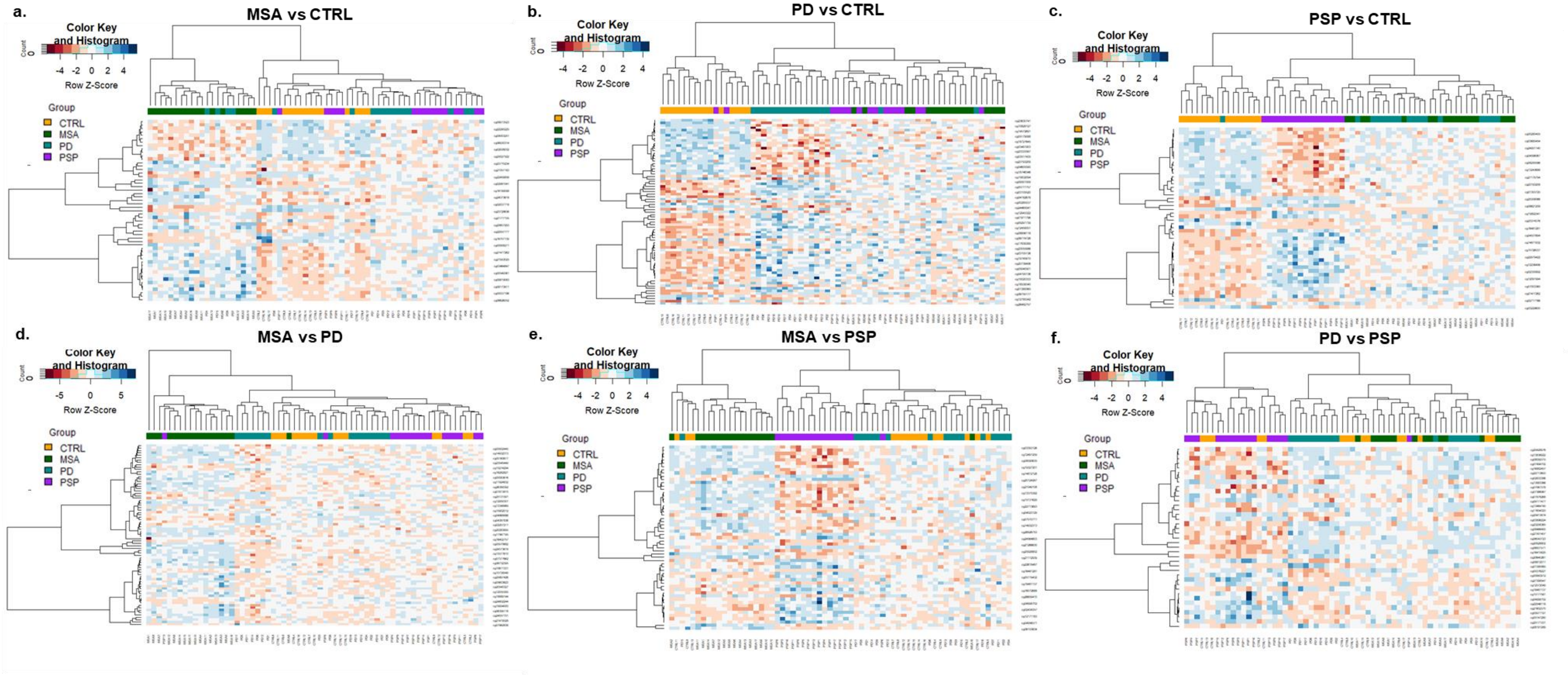

Supplementary fig. S3 Heatmap of the topmost significant differentially methylated loci ($p < 0.0001$) identified in (a.) MSA compared to controls (b.) PD compared to controls (c.) PSP compared to controls, (d.) MSA compared to PD, (e.) MSA compared to PSP, and (f.) PD compared to PSP. The rows represent CpGs, columns represent samples, and the colours represent the direction as well as the magnitude of effect (adjusted β values) in all the samples

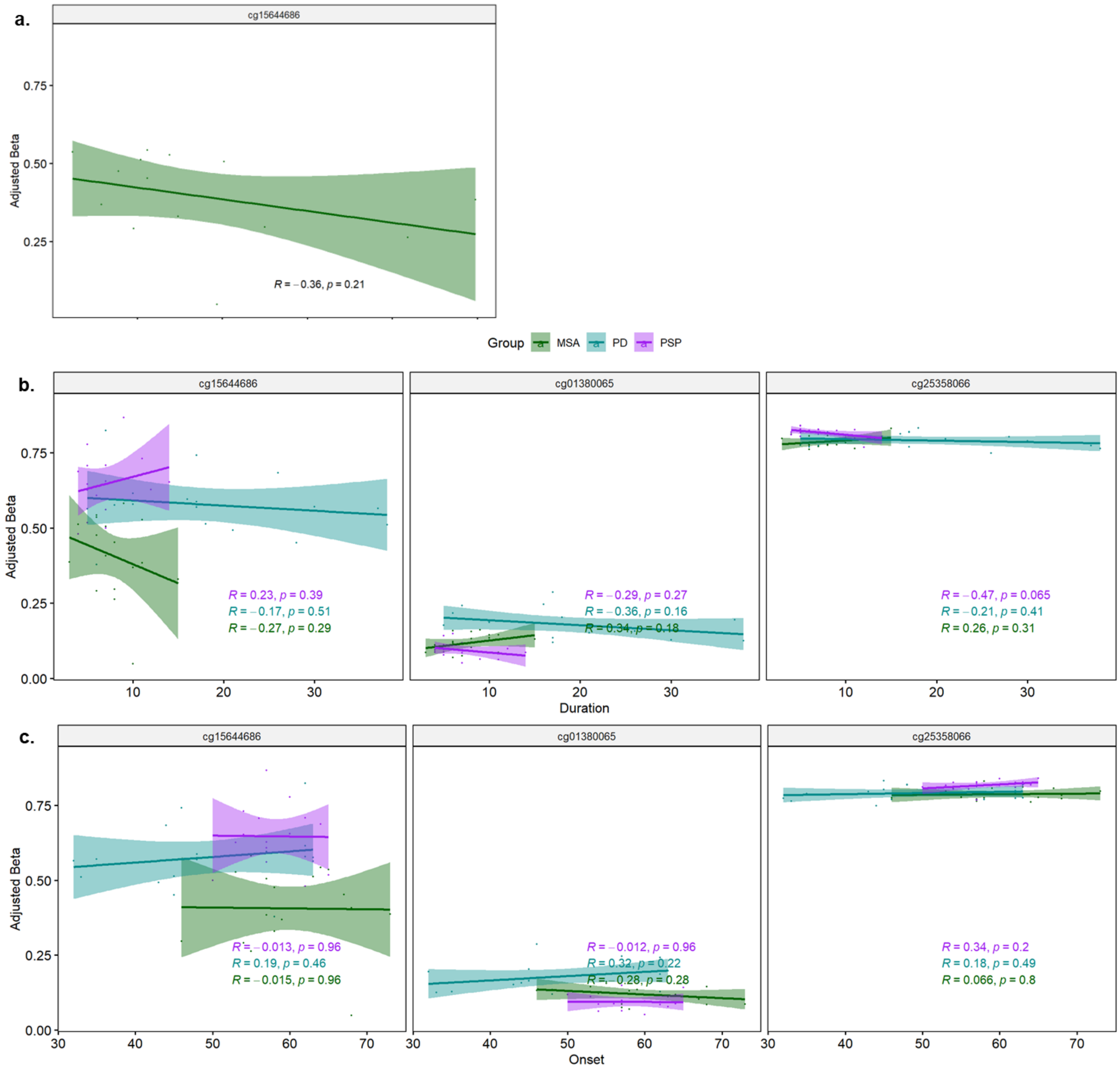

Supplementary fig. S4 Correlation between differential methylation levels and disease associated traits for the DMPs cg15644686 (*BCL7B*), cg01380065 (*UBE2F*), and cg25358066 (*D2HGDH*) in the different disease comparisons. Scatter plot and trend line (Pearson's correlation) showing correlation between methylation levels and (a) average GCI, (b) disease duration, and (c) disease onset. MSA – multiple system atrophy (mixed subtype), PD – Parkinson's disease, PSP – progressive supranuclear palsy, AvgGCI – average number of glial cytoplasmic inclusions

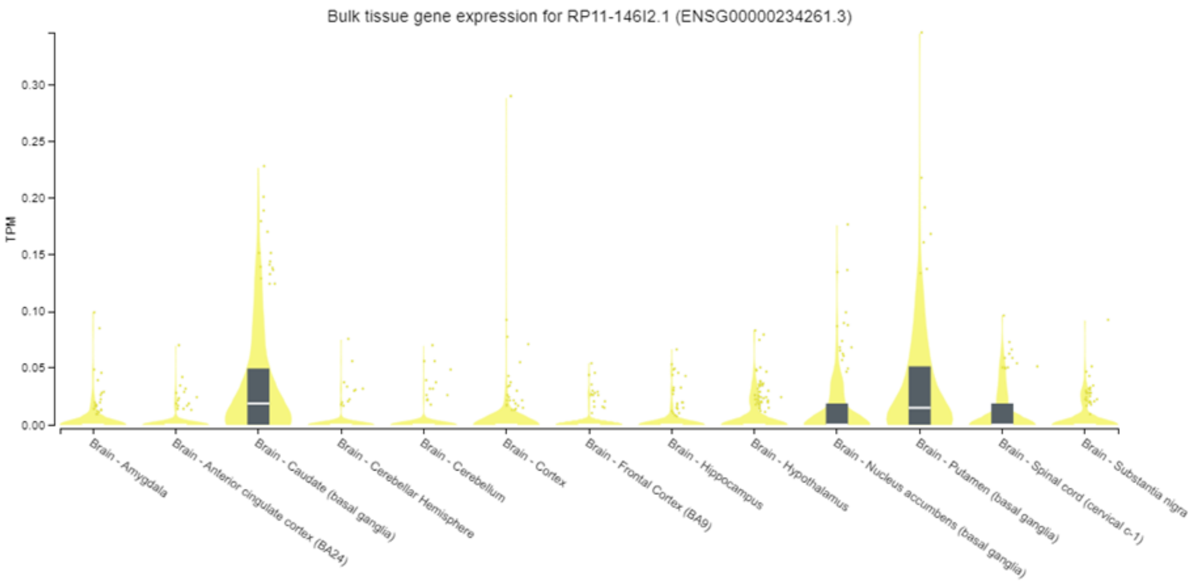

Supplementary fig. S5 Bulk tissue expression levels for the lincRNA encoded by the novel transcript ENSG00000234261 (where the CpG cg15274294 maps to) in different regions within the healthy brain

Gene expression data from GTEx analysis release V8 obtained from the GTEx Portal on 01/03/24 (https://www.gtexportal.org/home/gene/ENSG00000234261).

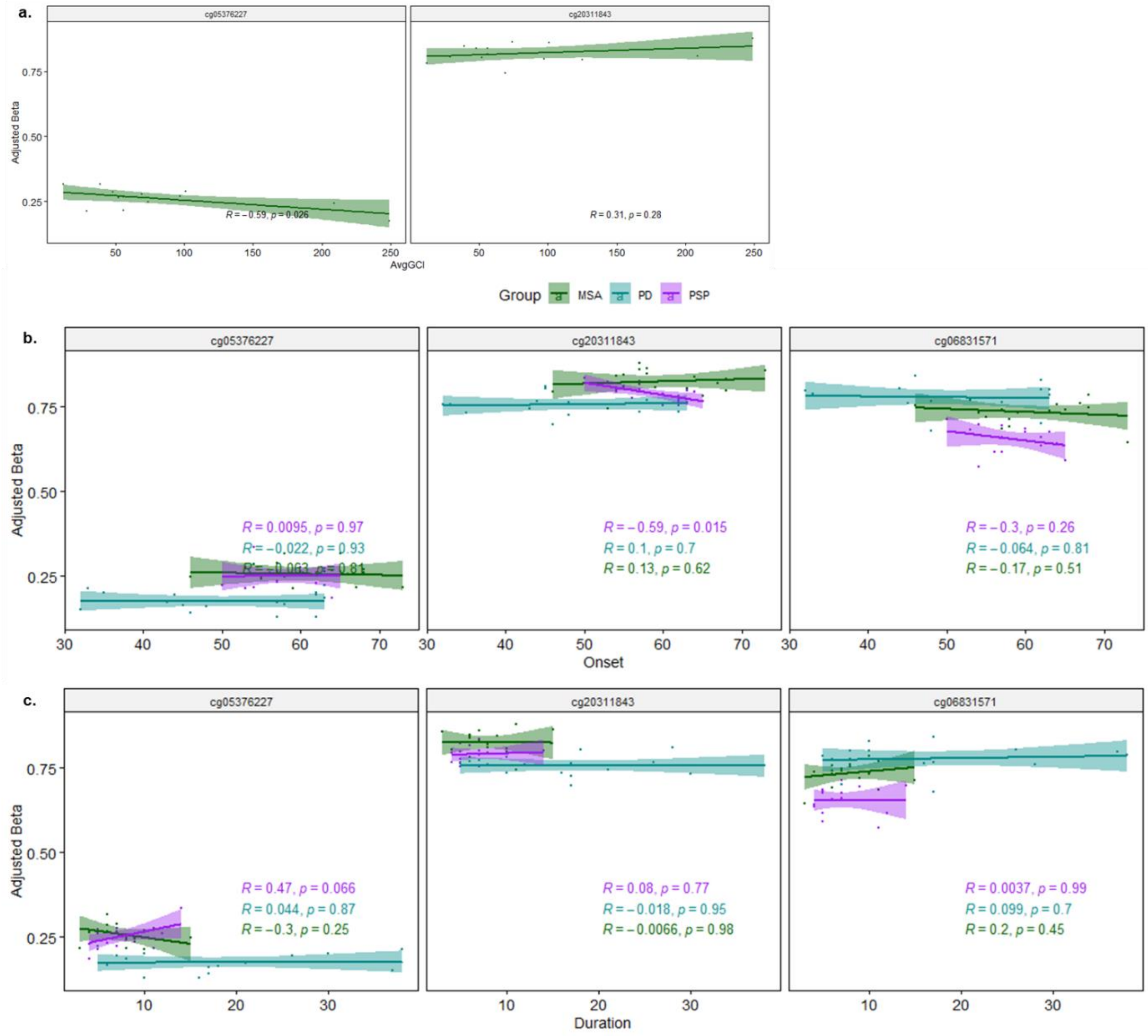

Supplementary fig. S6 Correlation between differential methylation levels and disease associated traits for the DMPs cg05376227 (*FMO6P*), cg20311843 (*OR51A7*) in MSA vs PD and cg06831571 (Chr11 – IGR) in PD vs PSP; scatter plot and trend line (Pearson's correlation) showing correlation between methylation levels and (a) average GCI, (b) disease onset, and (c) disease duration. MSA– multiple system atrophy (mixed subtype), PD – Parkinson's disease, PSP – progressive supranuclear palsy, AvgGCI – average number of glial cytoplasmic inclusions

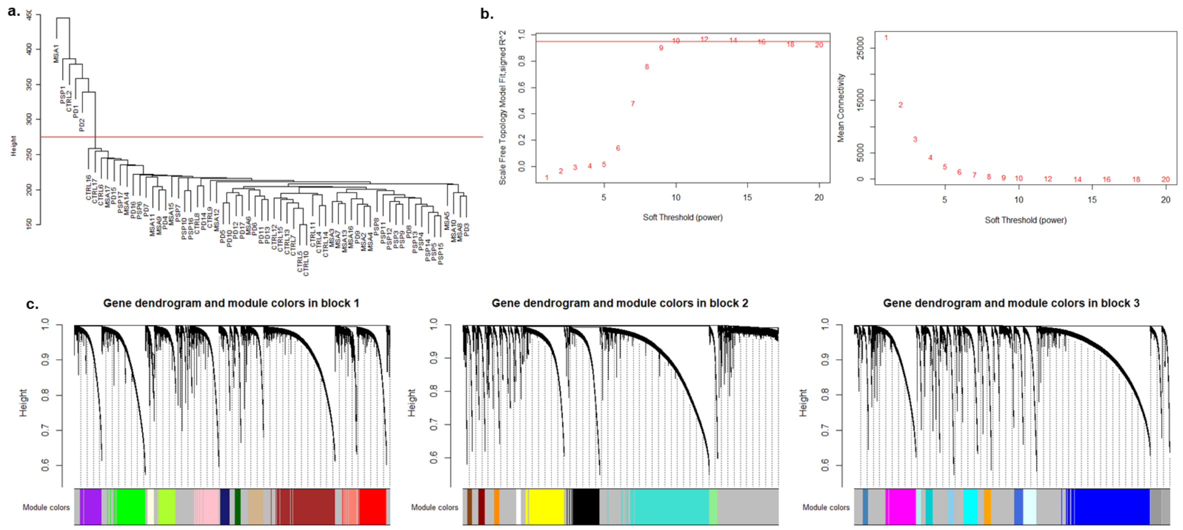

Supplementary fig. 7 Cluster trees and scale-free topology criteria for the comethylation network: (a) Sample clustering to detect outliers, (b) scale-free topology (SFT) plot for choosing the soft-thresholding power β for the signed weighted correlation network; left: SFT index R^2 (y-axis) as a function of different powers β (x-axis), right: mean connectivity (y-axis) as a function of the power β (x-axis), (c) hierarchical clustering dendrogram generated using blockwise module detection

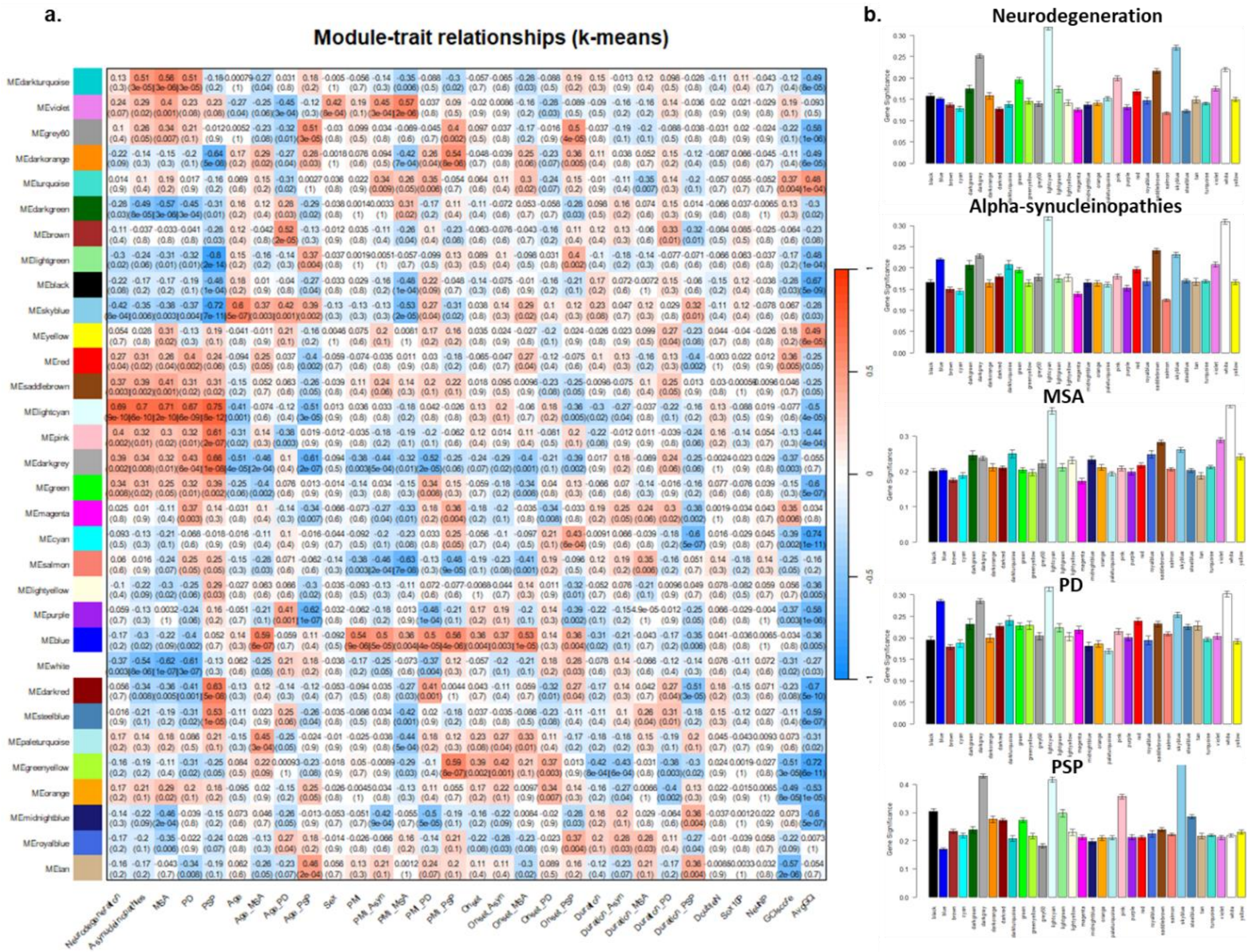
a.
Module-trait relationships (k-means)
MEdarkturquoise
MEviolet
MEgrey60
MEdarkorange
MEturquoise
MEdarkgreen
MEbrown
MElightgreen
MEblack
MEskyblue
MEyellow
MEred
MEsaddlebrown
MElightcyan
MEpink
MEdarkgrey
MEgreen
MEmagenta
MEcyan
MEsalmon
MElightyellow
MEpurple
MEblue
MEwhite
MEdarkred
MEsteelblue
MEpaleturquoise
MEgreenyellow
MEorange
MEmidnightblue
MEroyalblue
MEtan
Neurodegeneration
Asynucleinopathies
MSA
PD
PSP
Age
Age_MSA
Age_PD
Age_PSP
Sex
PMI
PMI_Asyn
PMI_MSA
PMI_PD
PMI_PSP
Onset
Onset_Asyn
Onset_MSA
Onset_PD
Onset_PSP
Duration
Duration_Asyn
Duration_MSA
Duration_PD
Duration_PSP
DodAtsN
SoxTP
NeuNP
GCIscore
AvgGCI
b.
Neurodegeneration
Alpha-synucleinopathies
MSA
PD
PSP
Gene Significance

Supplementary fig. 8 Module-trait correlations and gene significance (a) heatmap showing the module-trait correlations and p values for all disease associated clinical/pathological traits, (b) gene significance of the different modules in all three disease groups (neurodegeneration), in MSA and PD (α-synucleinopathies), and in the individual disease groups – MSA, PD, and PSP. MSA– multiple system atrophy, PD – Parkinson's disease, PSP – progressive supranuclear palsy

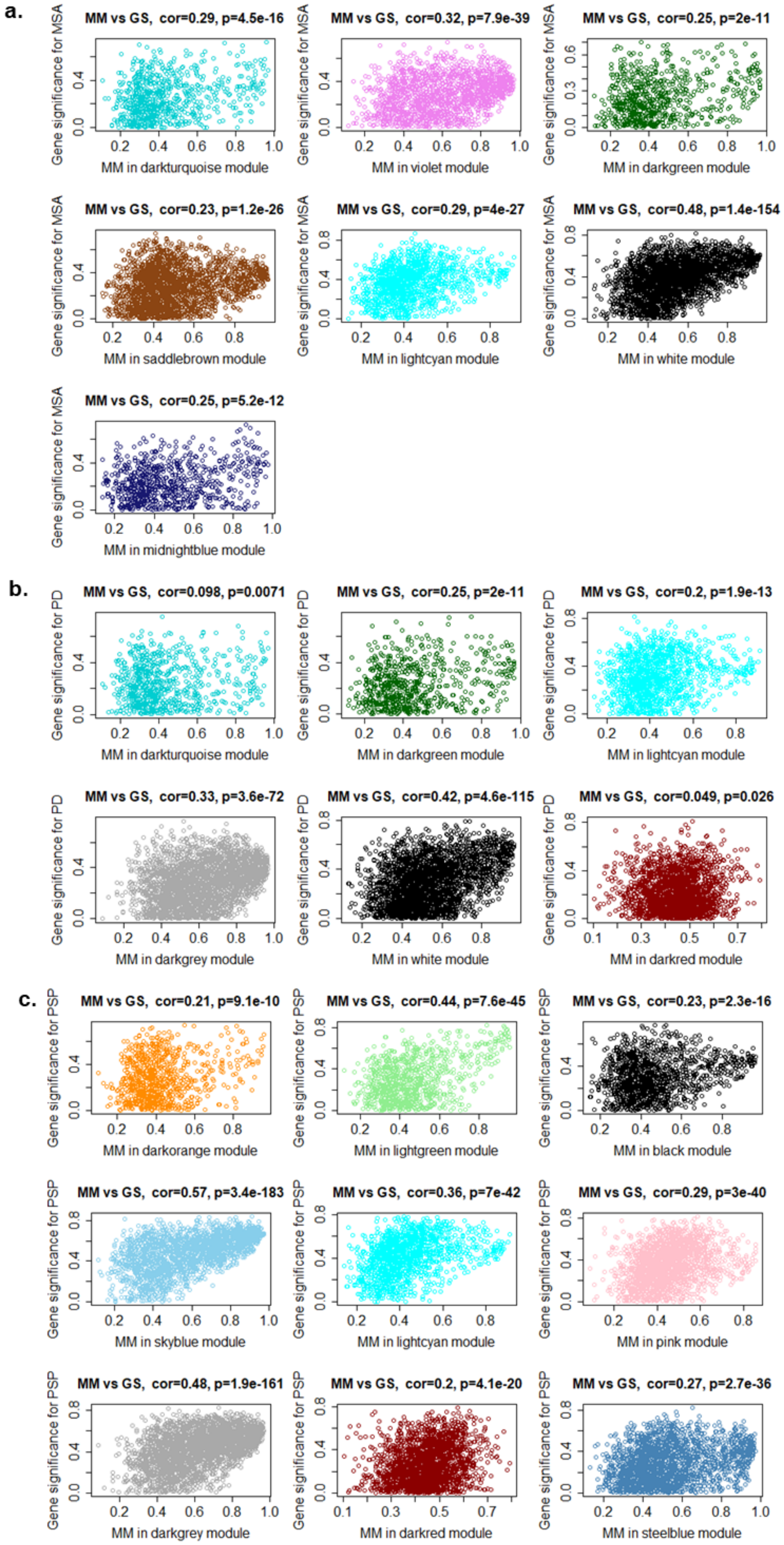
a.
MM vs GS, cor=0.29, p=4.5e-16
Gene significance for MSA
MM in darkturquoise module
MM vs GS, cor=0.32, p=7.9e-39
Gene significance for MSA
MM in violet module
MM vs GS, cor=0.25, p=2e-11
Gene significance for MSA
MM in darkgreen module
MM vs GS, cor=0.23, p=1.2e-26
Gene significance for MSA
MM in saddlebrown module
MM vs GS, cor=0.29, p=4e-27
Gene significance for MSA
MM in lightcyan module
MM vs GS, cor=0.48, p=1.4e-154
Gene significance for MSA
MM in white module
MM vs GS, cor=0.25, p=5.2e-12
Gene significance for MSA
MM in midnightblue module
b.
MM vs GS, cor=0.098, p=0.0071
Gene significance for PD
MM in darkturquoise module
MM vs GS, cor=0.25, p=2e-11
Gene significance for PD
MM in darkgreen module
MM vs GS, cor=0.2, p=1.9e-13
Gene significance for PD
MM in lightcyan module
MM vs GS, cor=0.33, p=3.6e-72
Gene significance for PD
MM in darkgrey module
MM vs GS, cor=0.42, p=4.6e-115
Gene significance for PD
MM in white module
MM vs GS, cor=0.049, p=0.026
Gene significance for PD
MM in darkred module
c.
MM vs GS, cor=0.21, p=9.1e-10
Gene significance for PSP
MM in darkorange module
MM vs GS, cor=0.44, p=7.6e-45
Gene significance for PSP
MM in lightgreen module
MM vs GS, cor=0.23, p=2.3e-16
Gene significance for PSP
MM in black module
MM vs GS, cor=0.57, p=3.4e-183
Gene significance for PSP
MM in skyblue module
MM vs GS, cor=0.36, p=7e-42
Gene significance for PSP
MM in lightcyan module
MM vs GS, cor=0.29, p=3e-40
Gene significance for PSP
MM in pink module
MM vs GS, cor=0.48, p=1.9e-161
Gene significance for PSP
MM in darkgrey module
MM vs GS, cor=0.2, p=4.1e-20
Gene significance for PSP
MM in darkred module
MM vs GS, cor=0.27, p=2.7e-36
Gene significance for PSP
MM in steelblue module

Supplementary fig. S9 Correlation between gene significance and module membership (kME) for modules significantly associated (adj.p≤0.001) with (a) MSA, (b) PD, and (c) PSP. MSA– multiple system atrophy, PD – Parkinson's disease, PSP – progressive supranuclear palsy, GS – gene significance, MM – module membership.

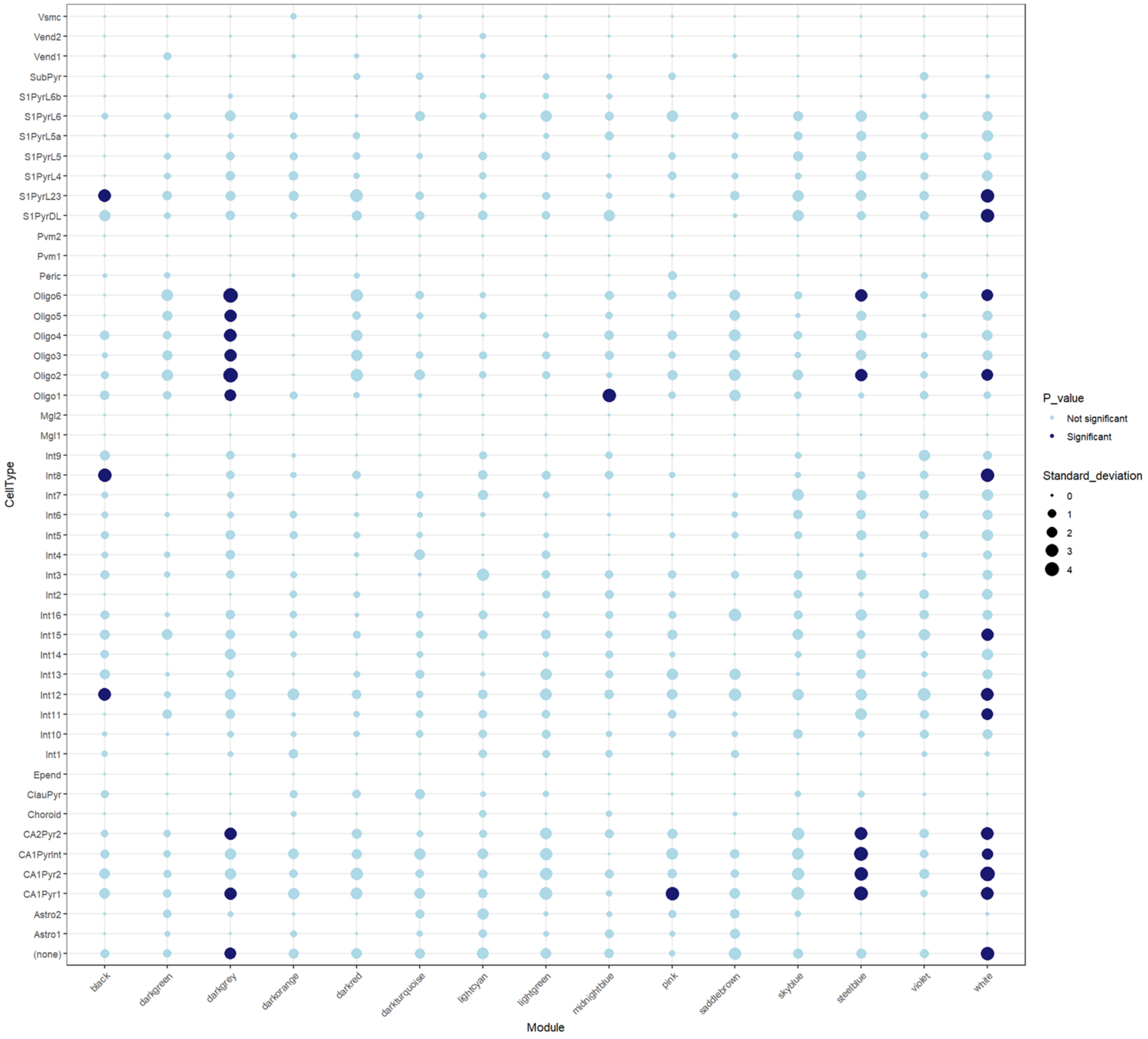

Supplementary fig. S10 Cell-type enrichment for the WGCNA modules associated with one or more disease groups. Enrichment for the different brain cell subtypes performed using the package EWCE and associated single-cell transcriptomic data which uses mouse to human homologs of genes associated with various cell types; dark blue circles represent significantly enriched cell types with adjusted $p < 0.05$ after Bonferroni corrections; the size of the circles represents the number of standard deviations (SD) from the mean

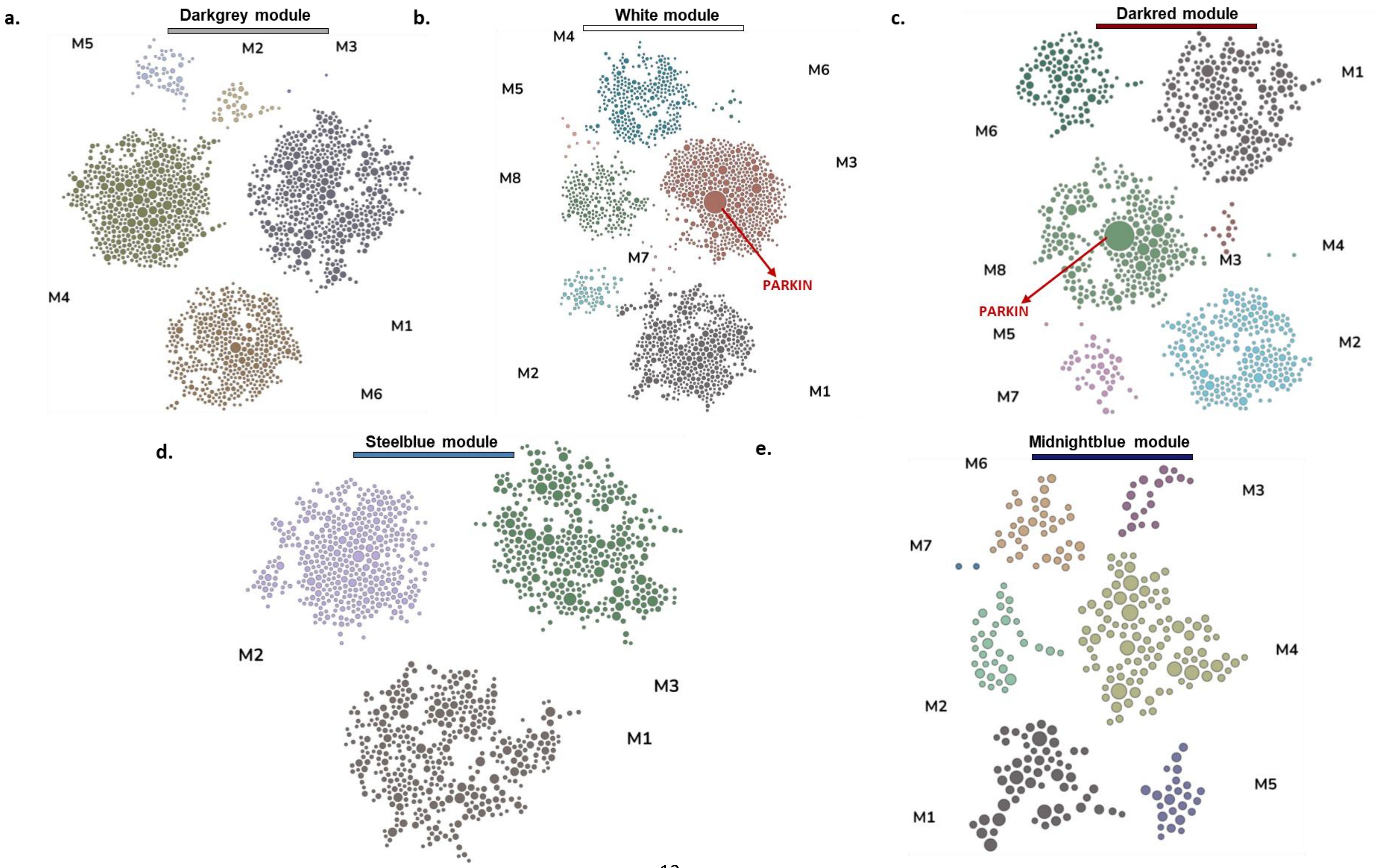
a.
Darkgrey module
M5
M2
M3
M4
M1
M6
b.
White module
M4
M6
M5
M3
M8
M7
PARKIN
M2
M1
c.
Darkred module
M1
M6
M4
M3
M8
PARKIN
M5
M2
M7
d.
Steelblue module
M2
M3
M1
e.
Midnightblue module
M6
M3
M7
M4
M2
M5
M1

Supplementary fig. 11 Frontal lobe specific functional network analysis on the oligodendrocyte-enriched modules created using HumanBase (https://hb.flatironinstitute.org/) for the (a) darkgrey module associated with all three disease groups (MSA, PD, PSP), (b) white module significantly associated with α-synucleinopathies (MSA and PD), (c) darkred and (d) steelblue modules negatively associated with α-synucleinopathies (MSA and PD) but positively associated with PSP, and (e) midnightblue module exclusively associated with MSA. Both white and darkred modules showed PARKIN as the hubgene in their submodules. MSA – multiple system atrophy, PD – Parkinson's disease, PSP – progressive supranuclear palsy
